# Crenigacestat, a selective NOTCH1 inhibitor, reduces intrahepatic cholangiocarcinoma progression by blocking VEGFA/DLL4/MMP13 axis

**DOI:** 10.1038/s41418-020-0505-4

**Published:** 2020-02-10

**Authors:** Serena Mancarella, Grazia Serino, Francesco Dituri, Antonio Cigliano, Silvia Ribback, Jingxiao Wang, Xin Chen, Diego F. Calvisi, Gianluigi Giannelli

**Affiliations:** 1 National Institute of Gastroenterology “S. de Bellis”, Research Hospital, Castellana Grotte, Italy; 2 Institute of Pathology, University of Regensburg, 93053 Regensburg, Germany; 3 Institute of Pathology, University of Greifswald, 17489 Greifswald, Germany; 4 Department of Bioengineering and Therapeutic Sciences and Liver Center, University of California, San Francisco, CA 94143 USA

**Keywords:** Cancer, Cancer models

## Abstract

Intrahepatic cholangiocarcinoma (iCCA) is a deadly disease with rising incidence and few treatment options. An altered expression and/or activation of NOTCH1–3 receptors has been shown to play a role in iCCA development and progression. In this study, we established a new CCA patient-derived xenograft model, which was validated by immunohistochemistry and transcriptomic analysis. The effects of Notch pathway suppression by the Crenigacestat (LY3039478)-specific inhibitor were evaluated in human iCCA cell lines and the PDX model. In vitro, LY3039478 significantly reduced Notch pathway components, including NICD1 and HES1, but not the other Notch receptors, in a panel of five different iCCA cell lines. In the PDX model, LY3039478 significantly inhibited the Notch pathway and tumor growth to the same extent as gemcitabine. Furthermore, gene expression analysis of iCCA mouse tissues treated with LY3039478 revealed a downregulation of VEGFA, HES1, and MMP13 genes. In the same tissues, DLL4 and CD31 co-localized, and their expression was significantly inhibited in the treated mice, as it happened in the case of MMP13. In an in vitro angiogenesis model, LY3039478 inhibited vessel formation, which was restored by the addition of MMP13. Finally, RNA-sequencing expression data of iCCA patients and matched surrounding normal liver tissues downloaded from the GEO database demonstrated that *NOTCH1*, *HES1*, *MMP13*, *DLL4*, and *VEGFA* genes were significantly upregulated in tumors compared with adjacent nontumorous tissues. These data were confirmed by our group, using an independent cohort of iCCA specimens. *Conclusion*: We have developed and validated a new iCCA PDX model to test in vivo the activity of LY3039478, demonstrating its inhibitory role in Notch-dependent angiogenesis. Thus, the present data provide new knowledge on Notch signaling in iCCA, and support the inhibition of the Notch cascade as a promising strategy for the treatment of this disease.

## Introduction

Cholangiocarcinoma (CCA) represents ~10–20% of newly diagnosed primary liver tumors worldwide, and despite its rarity, is the second most common malignant liver tumor after hepatocellular carcinoma (HCC) [1]. CCA is classified as peri-hilar (pCCA), distal (dCCA), and intrahepatic (iCCA) according to the anatomical site of origin. In the last decade, a global trend of increasing incidence and mortality by iCCA has been observed, which is in contrast with the decreasing trends of pCCA and dCCA [2]. This is due, at least partly, to the limited and largely ineffective treatment options. Surgical resection is currently the only means of radical treatment, but most of the patients are in the mid–late stage of the disease at the time of diagnosis. Consequently, this therapeutic option is limited only to ~20% of iCCA patients. Nevertheless, even after resection, the median time to disease-free survival is ~26 months, with recurrence rates of 60–65% [3, 4], due to factors including vascular invasion, lymph node metastasis, and cirrhosis. No systemic therapies have so far improved overall survival, with the combined administration of chemotherapeutic agents such as gemcitabine and cisplatin being of partial benefit. The lack of targeted therapies is mainly due to the poor knowledge of the molecular mechanisms underlying the development of iCCA, although robust experimental evidence suggests a central role for the Notch signaling in cholangiocarcinogenesis [5]. Specifically, altered expression and/or activation of the NOTCH1/2 receptors and the canonical ligand JAGGED1 indicate that the canonical Notch pathway is overactive during iCCA formation via HES1 [6]. In contrast, overexpression of NOTCH3 sustains iCCA cell survival through the activation of the PI3K/Akt cascade via a noncanonical mechanism independent of the recombination signal-binding protein for immunoglobulin Kappa J region (RBPJ) transcription factor [6].

At the molecular level, it is well understood that cleavage by γ-secretase, an intracellular proteolytic enzyme, releases the active fragment, known as “Notch Intracellular Domain” (NICD) from NoTCH receptors, which is bound to Jagged (Jagged1, 2) or Delta- like (Dll1, 3, and 4) ligands. Both receptors and ligands are cell surface transmembrane proteins, and therefore, Notch activation is initiated by direct cell–cell interactions. Once generated, NICD enters the cell nucleus and binds RBPJ, which acts as a transcriptional co-activator, by inducing important effects on cell fate, reprogramming, and growth [7]. Thus, it is not surprising that the gamma-secretase inhibitor (GSI) LY3039478, orally administered, is currently under investigation in clinical trials for different gastrointestinal advanced malignancies (https://clinicaltrials.gov/ct2/show/NCT02784795).

Moreover, iCCA is characterized by an intense desmoplastic reaction and a high intratumoral heterogeneity, mainly derived from the different origin of tumor cells such as hepatic stem/progenitor cells, periductal glands, malignant transformation of cholangiocytes, or even transdifferentiation of adult hepatocytes [5, 8, 9]. A tight interaction exists between iCCA and tumor microenvironment cells, including myofibroblasts, immune, and endothelial cells, soluble factors such as cytokines, and components of the extracellular matrix (ECM). This functional interplay among the various cell types results in the modulation of cellular communication, differentiation, invasiveness, chemoresistance, and epithelial-to-mesenchymal transition [10, 11].

In addition, neovascularization commonly occurs in iCCA, and plays a crucial role in tumor progression and dissemination [12–14], being the microvessel density an independent prognostic factor for this disease [13]. However, very little is known about the molecular interaction between cancer and endothelial cells. All these data together suggest that the crosstalk between tumor and the surrounding microenvironment is likely responsible for iCCA progression. Nevertheless, the molecular crosstalk between the Notch pathway and neovascularization has never been investigated to date. The aim of this study is to investigate the effectiveness and the mechanism of action of the GSI LY3039478 in preclinical experimental models of iCCA.

## Materials and methods

### Cell lines and reagents

The HUCCT1, RBE, KKU-M123, and KKU-M156 human iCCA cell lines and the KMCH1 HCC/iCCA mixed cell line were used in the experiments, and cultured as previously reported [15]. Cells, before use, were proven to be free of mycoplasma contamination via the MycoFluor^TM^Mycoplasma detection Kit (ThermoFisher Scientific, Waltham, MA). Human umbilical vein endothelial cells (HUVECs) were cultured and grown in EndoGRO-LS Supplement Kit (Merck Millipore, Burlington, MA). Cell lines and mice were treated with Crenigacestat (LY3039478, Selleckchem Chemicals, Houston, TX), a small molecule that prevents the release of NICD by inhibiting the proteolytic activity of the γ-secretase complex, thus reducing Notch signaling and downstream biological effects. Stock solutions were prepared at 10 mM in dimethylsulfoxide (DMSO, Invitrogen) and stored in aliquots at −20 °C. Gemcitabine (Selleck Chemicals) was used as a chemotherapeutic control in the in vivo model. The recombinant protein MMP13 (R&D System, Minneapolis, MN) was used at 50 ng/ml.

### Western blot analysis

Total proteins were extracted from cells and tissues using the T-PER Tissue Protein Extraction Reagent (ThermoFisher Scientific) with the Halt Protease & Phosphatase Inhibitor (ThermoFisher Scientific). Proteins were electrophoresed in 4–15% Tris-glycine sodium dodecyl sulfate-polyacrylamide gel (Bio-Rad Laboratories, Hercules, CA). Membranes were incubated with the following antibodies: human primary anti-Notch cleaved 1 (1:1000, Cell Signaling Technology, Pero, Italy); purified human anti-HES1 (1:1000, Cell Signaling Technology, Danvers, MA); anti-VEGFA (1:1000, Abcam, Cambridge, UK), anti-DLL4 (1:1000, Abcam, Cambridge, MA), anti-CD31 (1:1000, Abcam, Cambridge, MA), and anti-glyceraldehyde-3-phosphate dehydrogenase (GAPDH) (1:1000, Merck Millipore, Burlington, MA, USA). A secondary anti-rabbit or anti-mouse antibody (1:5000, Cell Signaling Technology, Danvers, MA) was used. Development was performed with a chemiluminescence system using the Clarity Max Western ECL Substrate (Bio-Rad Laboratories), and the signal displayed by the ChemiDoc MP instrument (Bio-Rad Laboratories) using Image Lab 5.2.1. The relative density of the bands was calculated using the Image J software.

### Immunohistochemistry and immunofluorescence

Immunohistochemistry and immunofluorescence were performed as previously described [16, 17]. In brief, for immunohistochemistry, sections were fixed in 4% paraformaldehyde, blocked with the 5% goat serum and Avidin–Biotin blocking kit (Vector Laboratories, Burlingame, CA), and incubated overnight at 4 °C with primary antibodies anti-CK-19, anti-CK-18, anti-CK-7 (1:50, Abcam, Cambridge, MA), anti-NOTCH1 (1:100, Lifespan Biosciences, Seattle, WA), anti-VEGFA (1:50, Abcam), anti-DLL4 (1:100, Abcam), anti-MMP13 (1:50, Proteintech Group Inc., Rosemont, IL, USA), anti-Actin (1:50, Santa Cruz Biotechnology, Inc., Heidelberg, Germany), anti-Vimentin (1:50, Cell Signaling Technology, Danvers, MA), and anti-Ki-67 (1:50, ThermoFisher Scientific) proteins. All the antibodies used in the experiments were previously validated for immunohistochemistry by the producing companies. Subsequently, the slides were incubated in a solution of 3% hydrogen peroxide for 10 min, and then the biotin-conjugated secondary antibody was applied at a 1:500 dilution for 30 min at room temperature. The immunoreactivity was visualized with the Vectastain Elite ABC kit (Vector Laboratories, Burlingame, CA) and 3,3′diaminobenzidine tetrahydrochloride (DAB) as the chromogen applied as a 0.02% solution containing 0.005% H_2_O_2_ in 50 mM ammonium acetate citrate acid buffer (pH 6.0). Alternatively, Vector NovaRed (Vector Laboratories) was used as the chromogen. The sections were slightly counterstained with Mayer’s hematoxylin, mounted, and analyzed using the Eclipse Ti2 microscope (Nikon Inc., Melville, NY). Image merge was performed using the Image J analysis software. As negative control, the slides were stained by omitting the primary antibody.

Immunofluorescence was performed as previously reported [17]. Specifically, sections were incubated with anti-MMP13 (1:50, Abcam), anti-DLL4 (1:50, Santa Cruz Biotechnology Inc.), and anti-CD31 (1:50, Abcam) antibodies, and after washing they were incubated with secondary goat anti-mouse or goat anti-rabbit immunoglobulin G H&L (Alexa Fluor 555 and Alexa Fluor 488, respectively, ThermoFisher Scientific).

### Establishment of the patient-derived xenograft (PDX) model

The tumor mass, immediately after being explanted from the patient, was collected and transferred to Hank's Balanced Salt Solution, then cut using a sterile scalpel in pieces smaller than 1 cm, and collected in cryovials to be frozen directly at −80 °C and moved after a few days in liquid nitrogen. The development of the PDX model, after approval of the Ethical Committee (Prot. N. 254/C.E), was conducted at the Biogem Animal House in Ariano Irpino (Avellino, Italy), in accordance with the National Academy of Sciences guidelines. Tissue fragments were implanted subcutaneously in the flanks of 4–5-week-old CD1 immunodeficient nude female mice. Each mouse was given drinking water ad libitum and a complete pellet diet (GLP 4RF21, Mucedola) during the study. Mice were monitored daily for clinical signs and mortality, and BW records were assessed weekly. Tumor growth was controlled every 2 weeks with Mitutoyo forceps. Experiments ended 8 weeks after the tumor implant, sacrificing animals with tumor masses greater than 15% of body weight (BW) and/or with a body weight loss (BWL) of 10%. All animals were weighed every 2–3 days during the experimental period. The BWL was determined as follows: body weight loss percent (% BWL max) = 100 − (mean BW day *x*/mean BW day 1 × 100), where BW*x* is the mean BW at the day of maximal loss during the experiment, and BW1 is the mean BW on the first day of the experimental period. At the end of the study, the mice were sacrificed by cervical dislocation, and the tumor masses were photographed and collected. Biopsies of 100 mm^3^ were implanted, and after a week from implantation, to allow the engraftment of the masses, the mice were divided into groups of ten animals and treated as follows: (1) vehicle only; (2) LY3039478 (8 mg/kg); (3) gemcitabine as a chemotherapeutic control (125 mg/kg). The Tumor Volume formula (mm^3^) = [length (mm) × width (mm)^2^]/2 was used, where width and length are the shortest and longest diameters.

### RNA extraction

Total RNA isolation was performed using TRIzol® (ThermoFisher Scientific) in combination with the TissueLyser homogenizer (Qiagen, Hilden, Germany), according to the manufacturer’s instructions. RNA concentration and quality were determined with the NanoDrop Spectrophotometer (ThermoFisher Scientific) and the Agilent 2100 Bioanalyzer (Agilent Technologies, Palo Alto, CA), respectively. Specifically, RNA with 260/280-nm ratios ≥1.8 and RIN ≥8 has been used for the analyses.

### Gene expression analysis

Gene expression analysis was performed using the Affymetrix microarray technology. Briefly, 100 ng of total RNA was reverse transcribed and labeled using the GeneChip™ WT PLUS Reagent Kit (Affymetrix, Santa Clara, CA) according to the manufacturer’s instructions. The biotinylated DNA was then hybridized to the GeneChip Human Transcriptome Array 2.0 (Affymetrix), containing more than 2,86,263 full-length transcripts covering 44,699 coding genes and 22,829 noncoding genes selected from RefSeq, ENSEMBL, and GenBank Homo sapiens genome databases. Chips were washed, stained, and scanned on the Affymetrix Complete GeneChip® Instrument System (Affymetrix), generating digitized image and raw-intensity data. Microarray data are available under accession number GSE134114 at the Gene Expression Omnibus (http://www.ncbi.nlm.nih.gov/geo/).

### Quantitative reverse-transcription real-time PCR (qRT-PCR)

One microgram of total RNA from PDX tissues was reverse transcribed with I Script Reverse Transcription Supermix (Bio-Rad Laboratories) according to the manufacturer’s instructions. Comparative real-time PCR was performed in triplicate, including no-template controls. Relative expression was calculated using the 2^−ΔΔCt^ method. The primers used were as follows: MMP13 Human PrimePCR™ SYBR® Green Assay ID: qHsaCIP0026824 (Biorad); Hs_NOTCH1_2_SG QuantiTect Primer Assay ID: QT01005109; Hs_HES1_1_SG QuantiTect Primer Assay ID: QT00039648; Hs_DLL4_1_SG QuantiTect Primer Assay ID: QT00081004; Hs_GAPDH_1_SG QuantiTect Primer Assay ID: QT00079247 (Qiagen) and primer sequences for VEGFA: forward, 5′-CAGATGTCCCGGCGAAGA-3′; reverse, 5′-GAGGGCGAGTCCCAGGAA-3′. For human iCCA samples, mRNA expression of the genes of interest was detected by qRT-PCR using validated TaqMan Gene Expression Assays for human *NOTCH1* **(**Hs01062014_m1**)**, *NOTCH2* **(**Hs01050702_m1), *NOTCH3* (Hs01128537_m1), *NOTCH4* (Hs00965889_m1), *DLL4* **(**Hs00184092_m1), *HES1* (Hs00172878_m1), *VEGFA* (Hs00900055_m1), *MMP13* (Hs00942584_m1), *RBPJ* (Hs00794653_m1), and *β-actin* (Hs01060665_g1) genes (ThermoFisher Scientific). PCR reactions were performed with 100 ng of cDNA of the collected samples, using an ABI Prism 7000 Sequence Detection System with TaqMan Universal PCR Master Mix (Applied Biosystems). Cycling conditions were as follows: denaturation at 95 °C for 10 min, 40 cycles at 95 °C for 15 s, and then extension at 60 °C for 1 min. Quantitative values were calculated by using the PE Biosystems Analysis software and expressed as N target (NT). NT = 2^−ΔCt^, wherein the ΔCt value of each sample was calculated by subtracting the average Ct value of the target gene from the average Ct value of the *β-actin* gene.

### In vitro experiments

For knockdown studies in vitro, randomly selected HUCCT1 and RBE iCCA cell lines were transfected with small-interfering RNA (siRNA) against human *NOTCH1* (# s9633), *NOTCH2* (# s9637), *NOTCH3* (s9640), *NOTCH4* (# s9643), or scrambled siRNA (# s4390846, negative control, ThermoFisher Scientific) via using Lipofectamine™ RNAiMAX Transfection Reagent (ThermoFisher Scientific) according to the manufacturer’s instructions. Transient transfection experiments with the pT3-EF1α-dnRBPJ plasmid and corresponding empty vector were conducted in the HUCCT1 cell line using the Lipofectamine 2000 Reagent (ThermoFisher Scientific), following the manufacturer’s protocol. All experiments were repeated at least three times in triplicate.

### Statistical and bioinformatic analysis

For microarray analysis, the raw data were preprocessed with background correction and normalization with the Affymetrix Expression Console. The Affymetrix Transcriptome Analysis Console 4.0 was used to identify significant differentially expressed genes using the Limma eBayes method. The genes have been then selected according to the fold-change and *P* value ≤0.05 (with or without FDR-correction). In order to evaluate whether the LY3039478 treatment could influence a set of genes from a certain pathway, we applied preranked gene set enrichment analysis (GSEA) [18] using the hallmark gene sets of the Molecular Signatures Database [19] with 1000 permutations. We used FDR *q* value <0.25 to identify significant gene sets. The minimum and maximum criteria for selection of gene sets from the collection were 10 and 500 genes, respectively.

In addition, Ingenuity pathway analysis (IPA) software (Qiagen, USA) was applied to uncover the canonical pathways, upstream transcriptional regulators, biological processes, and molecular networks modulated from LY3039478 treatment in PDX mice. Hierarchical clustering was generated using the Alt Analyze 2.1.3 software [20]. Statistical analysis was performed using the GraphPad Prism 5.0 statistical software and Excel (Microsoft, Redmond, WA).

The statistical differences between different conditions were assessed with either two-tailed Student’s *t* test or Mann–Whitney test. All values were expressed as the mean ± SEM of data obtained from at least three independent experiments. The results were considered statistically significant with *P* < 0.05. RPBJ putative transcription factor binding motifs on DLL4, VEGFA, and MMP13 gene promoters were predicted using the Eukaryotic Promoter Database New (EPDnew; https://epd.epfl.ch) that combine EPD promoters with promoter-specific high-throughput data [21].

### Angiogenesis assay

Angiogenesis was assessed by seeding in pure Matrigel Matrix (Corning, NY) 1.4 × 10^4^ HUVECs per well in 100 μL of EndoGRO-LS Complete Culture Media Kit (Merck Millipore, Burlington, MA). At the same time, HUVECs were treated with DMSO (ctrl) or with LY3039478 or MMP13 recombinant protein 50 (ng/ml), or with a combination of LY3039478 and MMP13. The assay was monitored in time lapse for 10 h at 37 °C and 5% CO_2_ using an inverted bright field microscope equipped with an incubator (Nikon Inc.). The images of the wells were obtained in a ×4 magnification. For quantification, the branch point values and the total length of the capillary tube were determined by the Image J software. Each experiment was repeated three times.

### Human tissue specimens

Human iCCA samples were collected at the Medical University of Greifswald (Greifswald, Germany). Institutional Review Board approval was obtained at the local Ethical Committee of the Medical University of Greifswald (approval # BB 67/10). Informed consent was obtained from all individuals.

## Results

### LY3039478 inhibits γ-secretase and downstream Notch pathway in iCCA cell lines

**Fig. 1:**
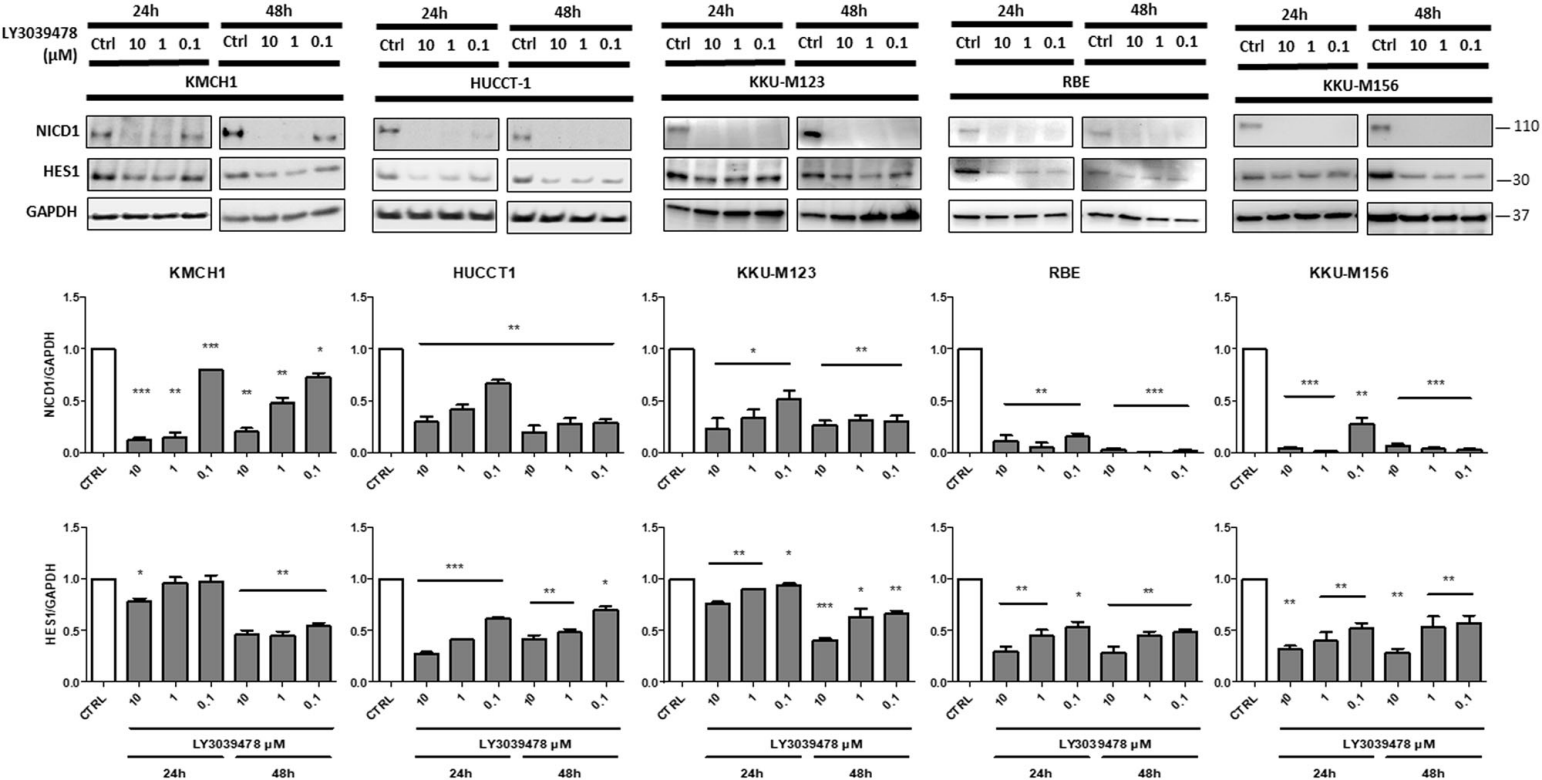
LY3039478 inhibits NOTCH1 and HES1 in iCCA experimental models. Western blot analysis demonstrates inhibition of NOTCH1 and HES1 on five iCCA cell lines treated for 24 and 48 h with LY3039478 even at low concentrations. Data are representative of three independent experiments. Histograms represent the mean ± SEM. *P < 0.05; **P < 0.01, ***P < 0.001 compared with treatment with vehicle (CTRL).

In order to investigate the effectiveness of LY3039478 on iCCA progression, we first examined its role in experimental cell culture models. Five iCCA cell lines (HUCCT1, KMCH1, RBE, KKU-M123, and KKU-M156) were tested for 24 and 48 h with different concentrations of LY3039478 (0.1–10 μM). In all cell lines, LY3039478 treatment significantly (*P* < 0.001) reduced the levels of activated NOTCH1 (NICD1) and those of Hairy and enhancer of split-1 (HES1) gene, a main target of the NOTCH signaling, when compared with vehicle (Fig. 1), in a dose-dependent manner. No reduction of the levels of the other NOTCH receptors (NOTCH2, 3, and 4) in cell lines treated with LY3039478 was observed (Supplementary Fig. 1), confirming LY3039478 as a NOTCH1- selective inhibitor.

### Establishment and characterization of a novel iCCA PDX model

To test LY3039478 effectiveness in vivo, we developed an iCCA PDX model. The generated PDX model showed an excellent proliferative capacity from day 21 after implantation, with no adverse effects on animal health (Supplementary Fig. 2A). Moreover, in all the monitored weeks the mice did not significantly change their BW (Supplementary Fig. 2B).

**Fig. 2:**
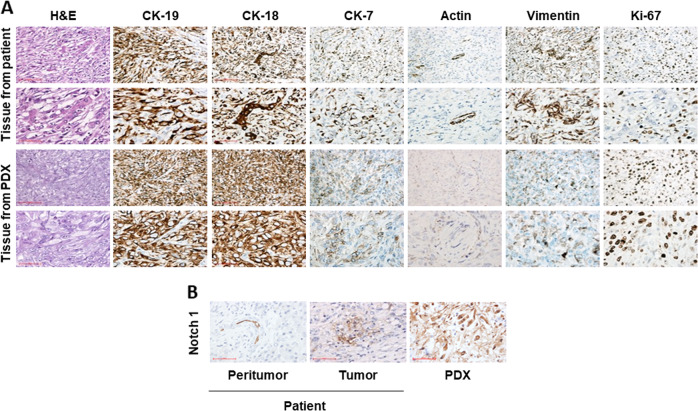
Immunophenotype characterization of patient and PDX iCCA tissues. **a** The immunohistochemical staining shows the same protein expression pattern of specific CCA markers such as cytokeratines and proliferation proteins both in PDX and in patient tissues. **b** The panel shows the increase of Notch1 immunoreactivity in the tumor tissue of the patient and the PDX, and a slight immunoreactivity in the peritumoral tissue, mainly localized in biliary vessels.

Subsequently, to prove that the PDX model reliably resembles the original human iCCA, we compared the histologic characteristics of the original human tumor and the related PDX. The expression of specific CCA markers, such as CK-19, CK-18, and CK-7, revealed a histological similarity between the original patient’s CCA tissue and the PDX (Fig. 2a). Moreover, the CCA lesions from the patient and the PDX were highly proliferative, as indicated by Ki67 staining. In addition, immunohistochemical staining (Fig. 2b) revealed overexpression of NOTCH1 in both PDX and tumor tissue, whereas no NOTCH1 immunoreactivity was detected in the surrounding normal tissue.

Overall, these data imply that the PDX model retains the phenotypic features of the original tumor tissue, thus confirming that the PDX model is a reliable system mimicking the original tumor.

### Gene expression confirms a strong correlation between the patient’s tumor mass and the PDX tumor tissue

To confirm similarities between the PDX and the original patient’s tumor mass, we also performed gene expression profile of the two entities by comparing them to the normal healthy liver tissue of the patient. Unsupervised hierarchical clustering analysis and principal component analysis of all probes clearly showed that the primary tumor sample and the PDX tissue clustered together while being completely separated from the normal healthy liver tissue (Supplementary Fig. 3A). This separation was further confirmed by unsupervised hierarchical clustering generated with deregulated genes between PDX tissue versus primary tumor and the normal healthy liver tissue, and by unsupervised hierarchical clustering generated with the top 2000 expressed genes (Supplementary Fig. 3B–D).

We also found a strong correlation of the degree of gene expression between the tumor mass from patient and the PDX tissue (Spearman *r* = 0.91; *P* < 0.0001, Supplementary Fig. 3E). Moreover, calculation of differentially expressed genes (fold change ≥2.0) between the patient tumor mass and the PDX tissue demonstrated that the two analyzed samples shared 99% of all genes (Supplementary Fig. 3F). Altogether, the present data indicate that the host mouse has a minimal effect on the molecular signature of the original tumor mass.

### LY3039478 effectiveness on iCCA in vivo

**Fig. 3:**
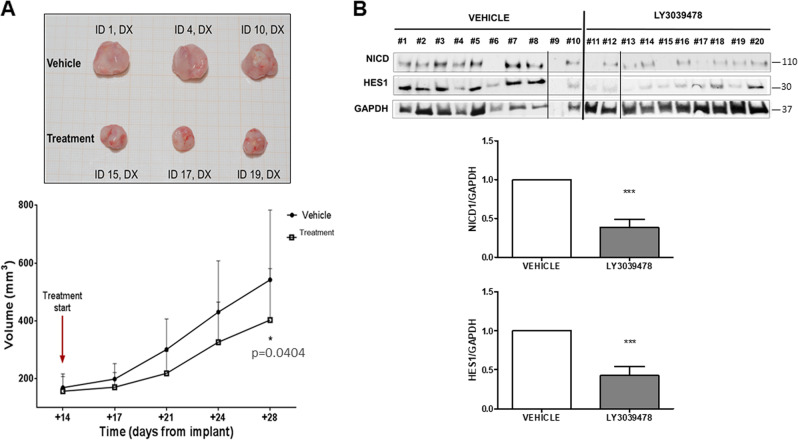
LY3039478 inhibits iCCA progression in the PDX model. **a** LY3039478 significantly (*P* < 0.05) inhibits tumor progression of iCCA in the PDX model. **b** Western blotting and semiquantitative evaluation by densitometry analysis of protein bands showing a significant inhibition of NICD1 and HES1 expression in treated PDX tissues (*P* < 0.001) compared with housekeeping GAPDH protein band for each tissue. The average value of NICD1 and HES1 levels among all mice treated with either LY3039478 or vehicle is reported in the graph versus vehicle treatment. PDX mice tissues, *n* = 10 for vehicle treatment, *n* = 10 for LY3039478 treatment.

To study the effectiveness of LY3039478 on the PDX model, mice were treated either with LY3039478 (8 mg/kg) or the drug vehicle. Tumor growth was monitored every 3 days from the first day of treatment. In mice that received the γ-secretase inhibitor, tumor growth was significantly reduced (*P* < 0.05) when compared with the vehicle at day +30. (Fig. 3a). Moreover, explanted tumors showed a significant reduction (*P* < 0.05) of activated NOTCH1 (NICD1) and HES1 protein levels in LY3039478-treated animals, but not in controls (Fig. 3b). These results strongly support the concept that LY3039478 inhibits the Notch pathway in vivo as observed in vitro, and is also responsible for reduction of in vivo tumor progression.

### LY3039478 inhibits tumor progression in the PDX iCCA model as gemcitabine

**Fig. 4:**
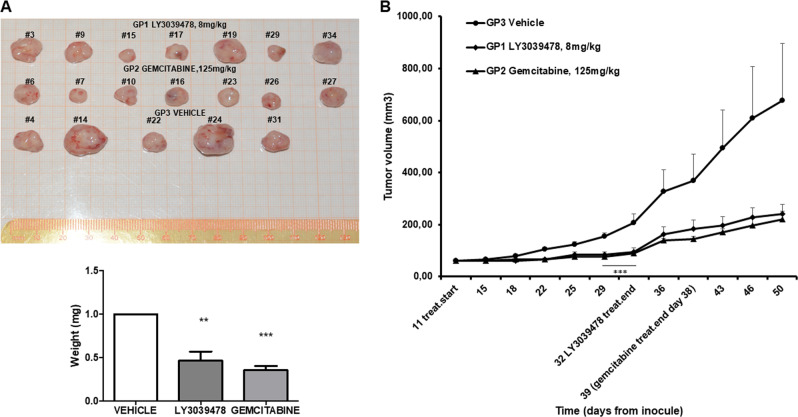
LY3039478 inhibits iCCA progression in the PDX model similarly to gemcitabine. The tumor progression of masses in PDX mice that received LY3039478 was delayed compared with vehicle and gemcitabine. Extra 18 days for LY3039478 group and extra 11 days for gemcitabine group after treatment end were given. The results show a slow recovery of progression in both treatment groups compared with the control group (vehicle) (**b**). At the end of the experiments, the size and weight (**a**) of the masses were visibly smaller than the control masses. **P < 0.01, ***P < 0.001 compared with treatment with vehicle.

To confirm our results, we repeated the experiment in vivo by adding a group of animals treated with gemcitabine, the standard-of-care chemotherapy for patients with advanced biliary tract cancer. In this new set of experiments, mice were randomized into three groups, respectively, treated with (1) vehicle; (2) gemcitabine at 125 mg/kg; (3) LY3039478 at 8 mg/kg. Consistently with our previous data, GSI significantly (*P* < 0.01) reduced iCCA progression, and its effectiveness was similar to that observed with gemcitabine. Moreover, the growth of iCCA PDX masses for additional 18 days for the LY3039478 group and 11 days for the gemcitabine group after the end of the treatment showed a slow recovery of progression in the two treatments cohorts when compared with the control group (vehicle) (Fig. 4a). At the end of the experiments, the size and weight of the masses were determined, and both treatment groups were found to be significantly (*P* < 0.01) smaller than the control tumors (Fig. 4b).

### Transcriptomic analysis in the PDX iCCA model and validation of gene expression

**Fig. 5:**
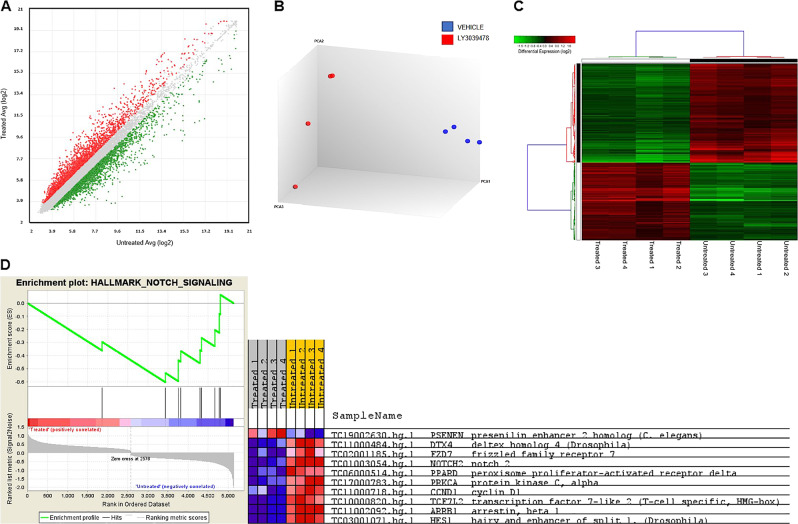
Global gene expression profile in PDX tissues treated and untreated with LY3039478. **a** Scatter plot of all assayed probes showing the distribution of differential expressed genes based on the expression data of treated and untreated mice. The *X* axis represents the averaged log2 signal of untreated samples, and the *Y* axis represents the averaged log2 signal of treated samples. **b** PCA and **c** unsupervised hierarchical clustering using differential expressed genes show a clear separation based on LY3039478 treatment. Each row represents a gene, and each column represents a sample. A color code represents the relative intensity of the expression signal, where red indicates high expression and green low expression, respectively, according to the scale shown at the top. **d** GSEA enrichment plot and heatmaps show an enrichment of gene signatures associated with Notch signaling.

To investigate in depth the molecular changes induced by LY3039478 in the PDX model, we performed gene expression analysis on the tissues from PDX mice subjected or not to LY3039478 treatment. Applying a cutoff threshold of *P* < 0.05 and fold change of ±1.5, we identified 2635 differentially expressed genes (1148 up- and 1487 downregulated, respectively) (Fig. 5a, Supplementary Table T1). PCA clearly showed the degree of separation between treated and untreated mice (Fig. 5b). This separation was further confirmed by unsupervised hierarchical clustering analysis (Fig. 5c).

In order to identify the potential pathways enriched in treated versus untreated PDX mice, we performed a GSEA (Supplementary Table T2). As shown in Fig. 5d, LY3039478-treated PDX mice were characterized by a low expression of the Notch pathway (normalized enrichment score = −18.727, NOM; *P* = 0.006 and FDR = 0.03).

The IPA upstream regulator analysis revealed a differential activation of several proteins depending on the treatment. One of the most significant upstream regulators predicted to be associated with deregulated genes was VEGFA. Indeed, the expression pattern of the deregulated genes after LY3039478 treatment revealed a strong correlation with VEGF signaling (−log *P* value 4.65E−04 and 5.41E−04), suggesting that angiogenesis is modulated by LY3039478 treatment. Nonetheless, the direct comparison between treatments revealed the degree of activation; indeed, in the PDX model VEGF was predicted to be inhibited (*z* score 1.69). Of note, two downregulated genes, namely *MMP13* and *HES1*, were found to be target molecules of the VEGF signaling pathway (Supplementary Fig. 4).

To validate the microarray results, we performed quantitative real-time PCR (qRT-PCR) for *HES1*, *MMP13*, and *VEGFA* on all PDX mice treated and untreated with LY3039478. All analyzed genes were significantly (*P* < 0.05, *P* < 0.01, and *P* < 0.001, respectively) downregulated after treatment, thus confirming the microarray data (Supplementary Fig. 5).

### LY3039478 inhibits angiogenesis via MMP13 in intrahepatic CCA

**Fig. 6:**
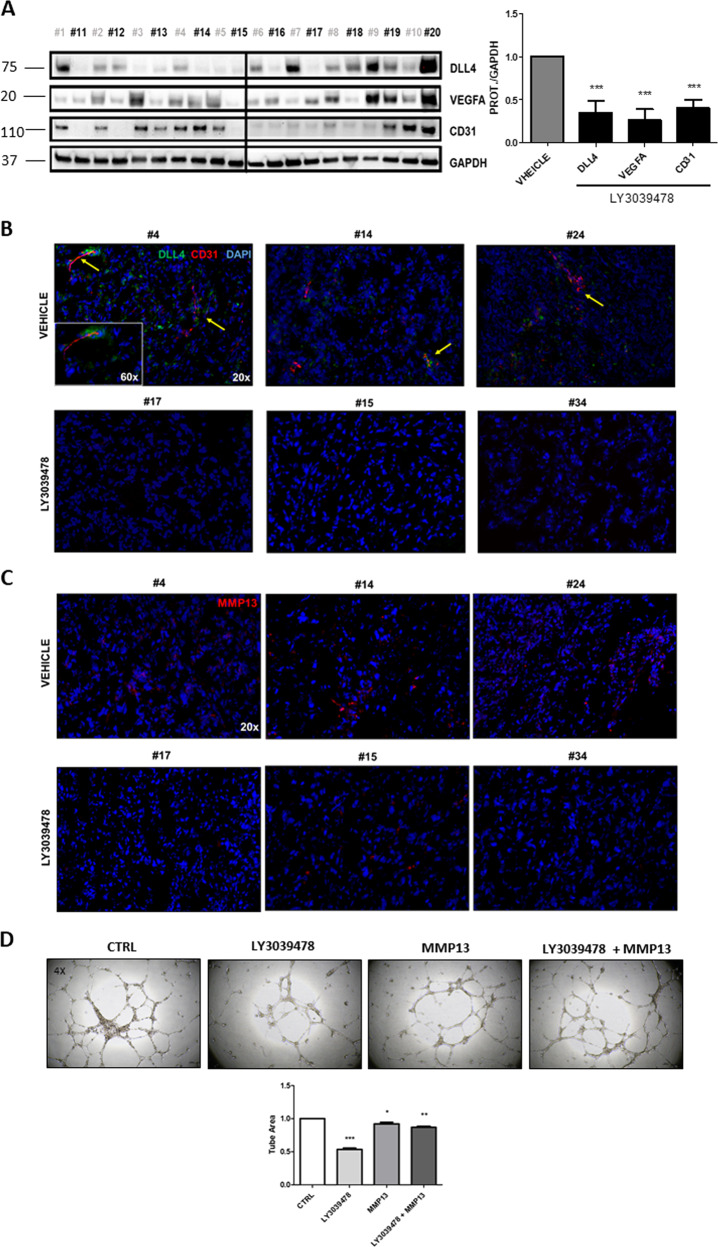
LY3039478 inhibits VEGFA, CD31, and DLL4 expression in the iCCA PDX model. **a** Western blot analysis and semiquantitative evaluation of DLL4, VEGFA, and CD31 expression in PDX mice tissues by densitometry analysis of protein bands reveals a downregulation of DLL4, VEGFA, and CD31 protein expression in PDX mice treated with GSI. The bands were measured compared with the housekeeping GAPDH protein band, for each tissue. Average value of DLL4, VEGFA, and CD31 expression levels among all mouse treated with LY3039478 or vehicle is reported in the graph. *P* value showed versus vehicle treatment. Tissues PDX mice *n* *=* 10 for vehicle treatment in gray, *n* *=* 10 for LY3039478 treatment in black. **b** Representative images with immunofluorescence staining show DLL4 and CD31 downregulation in representative images of PDX tissues treated with LY30349478. DLL4 (green) and CD31 (red) and overlapping staining (yellow) were immunolocalized in PDX tissues. The yellow arrows highlight the detail of the co-localization of DLL4 and CD31 in PDX tissues (#4, #14, #24) not treated with LY339478. DAPI, 4′,6‐diamidino‐2‐phenylindole. **c** Immunofluorescence staining with MMP13 in red and nucleus in DAPI shown a significantly reduction of MMP13 in iCCA PDX tissues treated with LY3039478. Magnifications: ×20; inset ×60. **d** Representative images demonstrate a significant (*P* < 0.001) destruction of the network created by the HUVECs following the treatment with LY3039478 (1 µM). The concomitant administration of MMP13 counteracts significantly (*P* < 0.01) drug effectiveness.

Based on IPA prediction, we hypothesized that LY3039478 inhibits iCCA tumor progression by hampering angiogenesis. To test our hypothesis, we investigated the effectiveness of LY3039478 on angiogenesis in vivo. Specifically, VEGFA, DLL4, and CD31 levels were significantly (*P* < 0.05) downregulated as measured by western blotting (Fig. 6a). Also, VEGFA colocalizes with CD31, and both expression as well as that of DLL4 was inhibited in the iCCA tissues from animals treated as compared with those untreated (Fig. 6b and Supplementary Figs. 6, 7). In addition, MMP13 immunolocalized by immunofluorescence was downregulated in treated versus untreated mice (Fig. 6c and Supplementary Figs. 8, 9). These results suggest that the treatment of LY3039478 induces the inhibition of the DLL4/Notch signaling, producing a deregulation of VEGFA, CD31, MMP13, and tumor vascularization.

To better investigate the functional role of LY3039478 in vitro, we challenged vessel formation by HUVECs with the GSI. After 18 h of treatment, LY3039478 blocked angiogenesis (Fig. 6d and Supplementary Video V1-4). However, the supplementation of the recombinant MMP13 protein to the assay reverted drug effectiveness, suggesting that blood vessel formation requires MMP13 activity.

### Validation of DLL4, VEGFA, and MMP13 genes as direct NOTCH targets

To determine whether DLL4, VEGFA, and MMP13 are NOTCH direct transcriptional targets, we performed an in silico prediction of the binding sites for RPBJ, the transcriptional co-activator of NOTCH [7], on *DLL4*, *VEGFA*, and *MMP13* gene promoters using the EPDnew database [21]. Noticeably, we identified several putative binding sites for RPBJ on *DLL4*, *VEGFA* and *MMP13* gene promoter regions (*P* < 0.01; Supplementary Fig. 10). The hypothesis of DLL4, VEGFA, and MMP13 being specific effectors of the NOTCH-RBPJ was further investigated via two distinct silencing approaches. First, the HUCCT1 cell line was subjected to transient overexpression of the dominant negative form of RBPJ (dnRBPJ), which has been shown to effectively inhibit the transcriptional program of the canonical Notch cascade [22]. In accordance with in silico data, forced overexpression of RBPJdn resulted in the downregulation of *DLL4*, *VEGFA*, and *MMP13* mRNA levels in HUCCT1 cells (Supplementary Fig. 11A). Subsequently, *NOTCH1* was knocked down in HUCCT1 and RBE cells via specific small interfering RNA (siRNA). As expected, a marked decrease of *DLL4*, *VEGFA*, and *MMP13* expression was detected following siRNA-mediated silencing of the *NOTCH1* gene in the two cell lines (Supplementary Fig. 11B). Noticeably, ubiquitous downregulation of MMP13 occurred also in the same cell lines subjected to *NOTCH2–4* knockdown, whereas mRNA levels of *HES1*, *DLL4*, and *VEGFA* were heterogeneously affected by the suppression of the same NOTCH isoforms (Supplementary Fig. 12A–C). Altogether, the present data indicate that DLL4, VEGFA, and MMP13 are Notch pathway targets in iCCA.

### LY3039478 molecular targets in iCCA patients

**Fig. 7:**
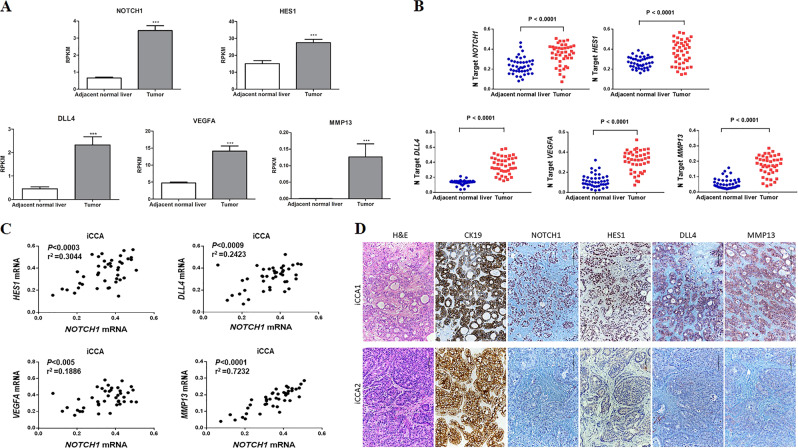
*NOTCH1*, *HES1*, *DLL4*, *VEGFA*, and *MMP13* mRNA expression in iCCA patients. **a** Analysis of 31 primary tumors from iCCA patients and matched surrounding normal liver tissues downloaded from the GEO database (GSE107943). Mean expression data were expressed in RPKM (Reads Per Kilobase Million). ***P < 0.001 calculated with Student’s *t* test. **b** NOTCH1 gene and its pro-angiogenic targets are overexpressed in human intrahepatic cholangiocarcinoma (iCCA). Levels of NOTCH1, DLL4, VEGFA, and MMP13 mRNA were significantly more elevated in iCCA (*n* = 42) than corresponding nontumorous surrounding livers (SL; *n* = 42), as detected by quantitative reverse-transcription PCR. Number target (NT) = 2^−ΔCt^, wherein ΔCt value of each sample was calculated by subtracting the average Ct value of the gene of interest from the average Ct value of the β-actin gene. Mann–Whitney test: vs SL, *P* < 0.0001. **c** Expression of the NOTCH1 gene correlates with mRNA levels of putative target genes (HES1, DLL4, VEGFA, and MMP13) in a collection of human intrahepatic cholangiocarcinoma (CCA) samples (*n* = 42). Linear regression analysis was used. **d** Representative expression patterns of CK19, NOTCH1, HES1, DDL4, and MMP13 in human intrahepatic cholangiocarcinoma (iCCA) as detected by immunohistochemistry. Upper panels: CCA case (CCA1) showing strong, concomitant immunoreactivity for NOTCH1, HES1, DDL4, and MMP13. Lower panels: CCA specimens (CCA2) exhibiting low levels of NOTCH1, HES1, DDL4, and MMP13. As expected, both iCCA display robust immunolabeling for CK19 (a biliary marker). Magnification: ×200; scale bar = 100 μm. H&E hematoxylin and eosin staining.

In order to validate most of the relevant preclinical data in patients with only iCCA, we analyzed RNA-sequencing expression data of 31 human iCCA specimens, and matched the surrounding normal liver tissues downloaded from the GEO database (GSE107943) [23]. Importantly, we found that *NOTCH1*, *HES1*, *DLL4*, *VEGFA*, and *MMP13* genes were significantly upregulated (*P* < 0.001) in tumors when compared with adjacent nonneoplastic tissues (Fig. 7a), confirming that LY3039478 molecular targets are indeed involved in iCCA pathogenesis.

**Fig. 8:**
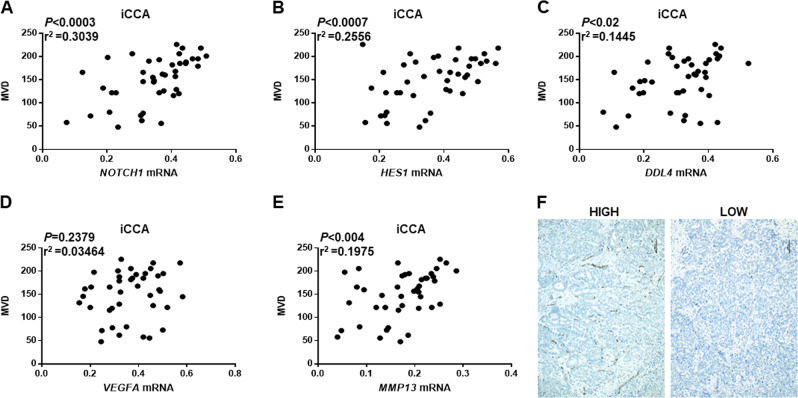
Relationship between microvessel density and NOTCH1, HES1, DLL4, MMP13, and VEGFA levels. Levels of tumor microvessel density (MVD) correlate with mRNA expression of NOTCH1 (**a**), HES1 (**b**), DLL4 (**c**), and MMP13 (**e**), but not with those of VEGFA (**d**), in a collection of human intrahepatic cholangiocarcinoma (iCCA) samples (*n* = 42). Linear regression analysis was used. **f** Representative examples of human iCCA specimens with high and low MVD.

To further substantiate these data, we evaluated the mRNA levels of *NOTCH1*, *HES1*, *DLL4*, *VEGFA*, and *MMP13* in our iCCA sample cohort (*n* = 42). We found that *NOTCH1*, *HES1*, *DLL4*, and *MMP13* expression was significantly higher in iCCA than in nontumorous surrounding livers (Fig. 7b). In addition, a significant, positive correlation between mRNA levels of *NOTCH1* and those of *HES1*, *DLL4*, *VEGFA*, and *MMP13* was identified (Fig. 7c). In addition, a statistically significant correlation (*P* < 0.02) of *NOTCH1*, *HES1*, *DLL4*, and *MMP13* mRNA levels with MVD was observed in the 42 iCCA tissues (Fig. 8a–e). No correlation was found with VEGFA, being the latter gene likely associated with other multiple genes, as predicted by IPA analysis.

Subsequently, we determined the protein levels of NOTCH1, HES1, DLL4, and MMP13 by immunohistochemistry in the same iCCA collection (*n* = 42; Fig. 7d). In particular, robust immunoreactivity for NOTCH1, HES1, DLL4, and MMP13 was detected in 76.2, 64.3, 59.5, and 71.4% iCCA specimens, respectively. Positive and equivalent immunolabeling for the four proteins was detected in iCCA cells as well as in the inflammatory tumor stroma (Supplementary Fig. 13A, lower panel). Faint/absent immunoreactivity was observed in normal healthy liver for the same proteins, except for biliary epithelial cells (Supplementary Fig. 13A, upper panel). Concomitant strong staining for the four proteins was observed in 25 of 42 (59.5%) iCCA. Among the remaining 17 iCCA specimens, simultaneous low levels of NOTCH1, HES1, DLL4, and MMP13 proteins were detected in 8 iCCA, showing the possibility of addressing a personalized treatment against these promising targets.

## Discussion

iCCA is a fatal disease and, although rare, is experiencing a growing incidence in North America and Europe. Treatment options are limited so that the prognosis of iCCA patients is extremely poor [24–26]. Consequently, the lack of clinically relevant tumor models drastically hinders the study of CCA and the development of highly predictive models of clinical outcomes. In order to overcome this obstacle, we generated an iCCA PDX model that maintains the key biological and molecular properties of the primitive tumor while remaining stable over the passages.

Previous evidence supports Notch signaling as a crucial pathway for cell fate decision during development and disease of several organs and cell types, including cholangiocarcinogenesis [27, 28]. Indeed, unrestrained activation of the Notch molecular cascade has been shown to be involved in the initiation and progression of iCCA [29], affecting tumor cell proliferation, survival, and migration [30]. Nowadays, a growing interest in the development of therapies that target the Notch signaling pathway at different levels of the cascade is emerging. Indeed, monoclonal antibodies, antisense or RNA interference and glycosylation/protease inhibitor strategies have been developed for NOTCH receptors and ligands [31]. Among these, GSI inhibitors are actively considered as cancer therapeutic options, based on the finding that the NOTCH1 signal inhibition in selective tumors might be curative [32]. Several GSI compounds have been investigated in numerous preclinical models and in a significant number of clinical trials as anticancer agents [33]. However, the toxicity of these GSI has hindered their use in the clinical practice.

In the present investigation, we evaluated the effect of the modulation of the Notch pathway using a new GSI, namely LY3039478, in iCCA. LY3039478 effectively reduced the levels of the Notch pathway components, including NICD1 and HES1 in cultured human CCA cell lines, while not affecting proliferation. Furthermore, in the iCCA PDX model ad hoc generated, we observed a significant delay in tumor growth without appreciable toxicity, when compared with vehicle and chemotherapy administration. The expression of NICD1, HES1, and DLL4 was reduced also in treated animals, supporting the notion that Notch inhibition by LY3039478 may reduce in vivo tumorigenesis. Importantly, we showed a downregulation of VEGFA and MMP13 in treated PDX tissues at the gene expression and protein level. Hosaka et al. [34] previously observed that HES1 induces the expression of *MMP13* and *VEGFA* genes by binding to three and two representative E-boxes, respectively, trans-activating them directly in the advanced phase of chondrocyte differentiation. However, this regulatory mechanism has not been investigated in other cellular environments or pathologies to date.

Further, it is important to note that the Notch signaling mediates direct cell-to-cell communication to establish differential cell processes in neighboring cells. This suggests the existence of dynamic relationships between Notch and its ligands on tumor cells and the surrounding nontumor cells in the microenvironment [35]. Several investigations have shown that the DLL4/Notch signaling pathway is essential to modulate angiogenic sprouting and blood vessel growth in close collaboration with VEGFA [36–38]. Our study in vivo highlights the importance of LY3039478 treatment as it inhibits not only the DLL4/Notch signaling but also the levels of MMP13, which promotes growth and tumor progression by degradation of the ECM [39]. MMP13, derived from stroma in the immediate vicinity of tumor cells, was identified along the invasion and metastasis of breast cancer, renal cell carcinoma, squamous cell carcinoma, and melanoma [40–43], supporting a crucial role of the microenvironment for tumor growth. The involvement of MMP13 in neovascularization of the iCCA was elucidated by the gain of function mediated by the recombinant MMP13 protein, which led to failed regression of vascular tube formation, despite the inhibition of DLL4/Notch signaling by LY3039478. Thus, we have demonstrated that the crucial contribution of MMP13 on tumor progression allows the maintenance of angiogenesis, which is indirectly blocked by LY3039478 through the inhibition of MMP13. Nonetheless, we cannot exclude additional, direct effects of MMP13 and the other Notch targets on tumor cells, as we showed positive immunoreactivity for HES1, DLL4, and MMP13 proteins in iCCA cells.

In conclusion, we have developed a new model of iCCA PDX that could be useful to test and develop new personalized therapeutic treatments in iCCA. Furthermore, we provide evidence on the activity of LY3039478 in mouse models in vivo, demonstrating that LY3039478 targets DLL4, VEGFA, and MMP13 proteins in iCCA. The importance of the NOTCH1/HES1/DLL4/VEGFA/MMP13 axis in cholangiocarcinogenesis was confirmed in a collection of human iCCA tumor patients, paving the path for further investigations into the role of these genes in this tumor type.

Thus, the present results provide support for testing LY3039478 in future cutting-edge clinical trials in iCCA.

## Supplementary information

Supplementary Table T1

Supplementary Table T2

SUPPLEMENTARY FIGURE LEGENDS

Supplementary Figure 1

Supplementary Figure 2

Supplementary Figure 3

Supplementary Figure 4

Supplementary Figure 5

Supplementary Figure 6

Supplementary Figure 7

Supplementary Figure 8

Supplementary Figure 9

Supplementary Figure 10

Supplementary Figure 11

Supplementary Figure 12

Supplementary Figure 13

Supporting information tables

Supplementary Video V1

Supplementary Video V2

Supplementary Video V3

Supplementary Video V4
