# Supplementary material for: Crenigacestat, a selective NOTCH1 inhibitor, reduces intrahepatic cholangiocarcinoma progression by blocking VEGFA/DLL4/MMP13 axis
Source: Cell Death Differ. 2020 Feb 10;27(8):2330–43. doi: 10.1038/s41418-020-0505-4 (PMC7370218; doi:10.1038/s41418-020-0505-4)
Supplement: Supplementary file 2 — Supplementary Table T2 [file 41418_2020_505_MOESM2_ESM.docx]

**Supplementary Table T2**

| **NAME** | **SIZE** | **ES** | **NES** | **NOM p-val** | **FDR q-val** |
| --- | --- | --- | --- | --- | --- |
| HALLMARK_MYOGENESIS | 28 | -0,59 | -2,56 | 0,000 | 0,000 |
| HALLMARK_APICAL_JUNCTION | 39 | -0,48 | -2,33 | 0,000 | 0,001 |
| HALLMARK_IL2_STAT5_SIGNALING | 29 | -0,39 | -1,72 | 0,002 | 0,067 |
| HALLMARK_TGF_BETA_SIGNALING | 18 | -0,51 | -1,99 | 0,002 | 0,023 |
| HALLMARK_EPITHELIAL_MESENCHYMAL_TRANSITION | 50 | -0,35 | -1,82 | 0,003 | 0,042 |
| HALLMARK_HYPOXIA | 37 | -0,41 | -1,99 | 0,004 | 0,029 |
| HALLMARK_PI3K_AKT_MTOR_SIGNALING | 20 | -0,47 | -1,84 | 0,005 | 0,042 |
| HALLMARK_NOTCH_SIGNALING | 10 | -0,60 | -1,89 | 0,006 | 0,038 |
| HALLMARK_XENOBIOTIC_METABOLISM | 19 | -0,48 | -1,87 | 0,007 | 0,037 |
| HALLMARK_ESTROGEN_RESPONSE_EARLY | 38 | -0,33 | -1,59 | 0,040 | 0,136 |
| HALLMARK_ADIPOGENESIS | 27 | -0,36 | -1,54 | 0,050 | 0,147 |

**Supplementary Table T2.** GSEA of differentially expressed genes betweeen LY3039478 treated versus untreated PDX mice. The significant hallmark based on NOM pvalue for differentially expressed genes. In the table is shown the name of the hallmark that is enriched, the normalized enrichment score (NES), the FDR q-value (FDR q-value).
