# Supplementary material for: Crenigacestat, a selective NOTCH1 inhibitor, reduces intrahepatic cholangiocarcinoma progression by blocking VEGFA/DLL4/MMP13 axis
Source: Cell Death Differ. 2020 Feb 10;27(8):2330–43. doi: 10.1038/s41418-020-0505-4 (PMC7370218; doi:10.1038/s41418-020-0505-4)
Supplement: Supplementary file 3 — SUPPLEMENTARY FIGURE LEGENDS [file 41418_2020_505_MOESM3_ESM.docx]

**SUPPLEMENTARY FIGURE LEGENDS**

**Supplementary Figure 1: LY3039478 does not inhibit NOTCH2, 3, 4 proteins in iCCA cell lines.** Western blot analysis demonstrates no inhibition of NOTCH2, 3, and 4 protein levels on five iCCA cell lines treated for 24 and 48h with LY3039478.

**Supplementary Figure 2: PDX model development.** Progression of tumor volume of masses (A) and body weight (B) of PDX mice with tumor masses from patient in two different re-implant passages.

**Supplementary Figure 3. Gene expression profile of the original tumor mass and the PDX tissue compared to the surrounding normal liver of the patient.** (A) Principal component analysis (PCA) and (B) unsupervised hierarchical clustering using all analyzed probes clearly showed that the primary tumor sample and the PDX tissue were in the same region but completely separated from the surrounding normal liver. (C) Unsupervised hierarchical clustering generated with deregulated genes between PDX mass versus primary tumor and the surrounding normal liver and (D) unsupervised hierarchical clustering generated with the top 2000 expressed genes. (E) Correlation gene expression profile among PDX mass and primary tumor. (F) Pie chart represents the percentage of shared genes and differential expressed genes between PDX mass and primary tumor.

**Supplementary Figure 4. Upstream regulators and their target molecules in the dataset predicted by IPA**. Genes in red denote upregulation and genes in green denote downregulation in response to the treatment based on data from microarray. Molecules in orange or in blue indicated predicted activation or inhibition, respectively. Lines in orange denote predicted activation; lines in blue - predicted inhibition; lines in yellow - findings inconsistent with the state of the downstream molecule; and lines in grey - effect not predicted.

**Supplementary Figure 5. Assessment of *HES1*, *VEGF* and *MMP13* genes in PDX tissues treated and untreated with LY3039478.** Real-Time PCR and the relative expression was normalized to the *GAPDH* housekeeping gene. All analyzed genes were significantly downregulated after treatment, confirming the microarray data. **P*< 0.05; ***P*<0.01, ****P*<0.001 compared to treatment with vehicle.

**Supplementary Figure 6. Immunofluorescence staining of CD31 and DLL4 in untreated PDX tissues untreated.** Co-localization of CD31 and DLL4 on untreated PDX tissues (#4, #14, #24), as determined by immunofluorescence. The 555-anti mouse IgG antibody and the 488-anti rabbit IgG antibody were used as secondary antibodies. Nuclei were stained with DAPI. Magnification: 20x; scale bar=100 μm.

**Supplementary Figure 7. Immunofluorescence staining of CD31 and DLL4 in PDX tissues treated with LY3039478.** Weak immunolocalization of CD31 and DLL4 on PDX tissues (#17, #15, #34) treated with LY3039478, as determined by immunofluorescence. The 555-anti mouse IgG antibody and the 488-anti rabbit IgG antibody were used as secondary antibodies. Nuclei were stained with DAPI. Magnification: 20x; scale bar=100 μm.

**Supplementary Figure 8. Immunofluorescence staining of MMP13 in untreated PDX tissues.** Immunolocalization of MMP13 in untreated PDX tissues (#4, #14, #24)**,** as determined by immunofluorescence. The 488-anti rabbit IgG antibody was used as secondary antibody. Nuclei were stained with DAPI. Magnification: 20x; scale bar=100 μm.

**Supplementary Figure 9. Immunofluorescence staining of MMP13 on PDX tissues treated with LY3039478.** Weak immunoreactivity for MMP13 in PDX tissues (#17, #15, #34) treated with LY3039478, as determined by immunofluorescence. The 488-anti rabbit IgG antibody was used as secondary antibody. Nuclei were stained with DAPI. Magnification: 20x; scale bar=100 μm.

**Supplementary Figure 10. *In-silico* prediction of RPBJ binding sites on DLL4, VEGFA and MMP13 genes promoters.** Bioinformatic identification of RPBJ binding sites on DLL4, VEGFA and MMP13 genes promoters through the EPDnew software, using a cut-off of *P*-value of 0.01. Binding sites are represented in red squares.

**Supplementary Figure 11.** Effect of inhibition of NOTCH transcriptional activity (A) and NOTCH1 expression (B) on the levels of HES1, DLL4, VEGFA, and MMP13 mRNA in the HUCCT1 intrahepatic cholangiocarcinoma (iCCA) cell line, as assessed by real-time RT-PCR. Equivalent data were obtained in RBE cells (not shown). (A) NOTCH transcriptional activity was hampered by the transient overexpression of a dominant negative form of the NOTCH transcriptional co-activator RBPJ (dnRBPJ). (B) NOTCH1 was silenced using a specific NOTCH1 siRNA. Forty-eight-hour time point after transient transfection and siRNA administration is shown. Data are means±SD of three experiments conducted in triplicate. Tukey-Kramer test: ** at least *P*<0.001.

**Supplementary Figure 12.** Effect of inhibition of NOTCH2 (A), NOTCH3 (B), and NOTCH4 (C) expression by specific on the levels of HES1, DLL4, VEGFA, and MMP13 mRNA in the HUCCT1 intrahepatic cholangiocarcinoma (iCCA) cell line, as assessed by real-time RT-PCR. Equivalent data were obtained in RBE cells (not shown). Forty-eight-hour time point after siRNA administration is shown. Data are means±SD of three experiments conducted in triplicate. Tukey-Kramer test: ** at least *P*<0.001.

**Supplementary Figure 13.** (A) Representative immunohistochemical patterns for NOTCH1, HES1, DLL4, and MMP13 proteins in human normal liver (upper panel) and intrahepatic cholangiocrcinoma (iCCA) specimens (lower panel). In normal livers, immunoreactivity for the four proteins is generally limited to biliary epithelial cells (black arrows) and few immune cells (red arrow). In iCCA, tumor cells are strongly positive in the cytoplasm and/or nucleus for anti-NOTCH1, -HES1, -DLL4, and -MMP13 antibodies. In addition, inflammatory cells (red arrows) and endothelial cells (not shown) exhibit variable degrees of immunoreactivity for the same proteins. (B) Strong immunoreactivity for MMP13 in an iCCA (upper panel, positive control), which disappears when omitting the primary antibody (lower panel, negative control) in the immunohistochemical reaction. Magnification: 200x in A; 100x in B. Scale bar = 100 μm.
