# Supplementary figures and images for: Crenigacestat, a selective NOTCH1 inhibitor, reduces intrahepatic cholangiocarcinoma progression by blocking VEGFA/DLL4/MMP13 axis
Source: Cell Death Differ. 2020 Feb 10;27(8):2330–43. doi: 10.1038/s41418-020-0505-4 (PMC7370218; doi:10.1038/s41418-020-0505-4)

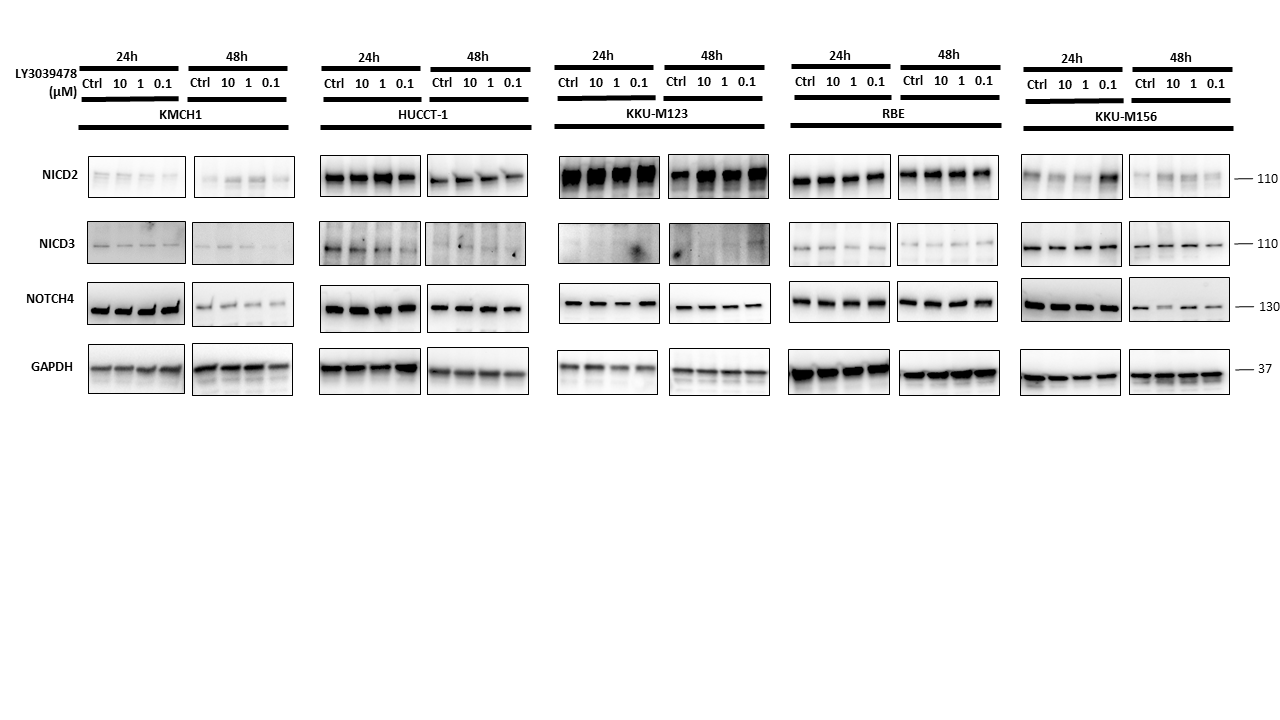

Supplement: Supplementary file 4 — Supplementary Figure 1 [file 41418_2020_505_MOESM4_ESM.tif]

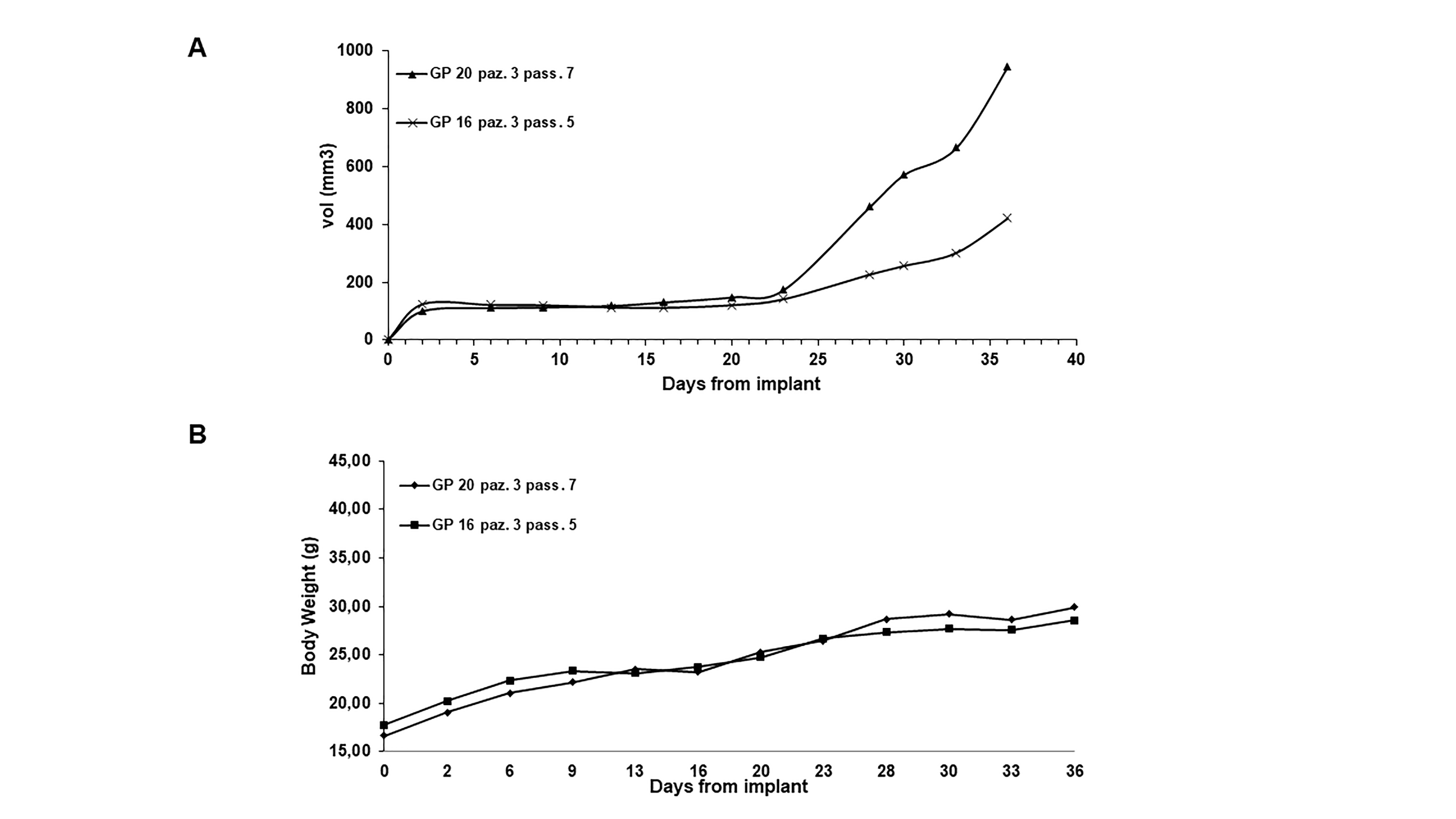

Supplement: Supplementary file 5 — Supplementary Figure 2 [file 41418_2020_505_MOESM5_ESM.tif]

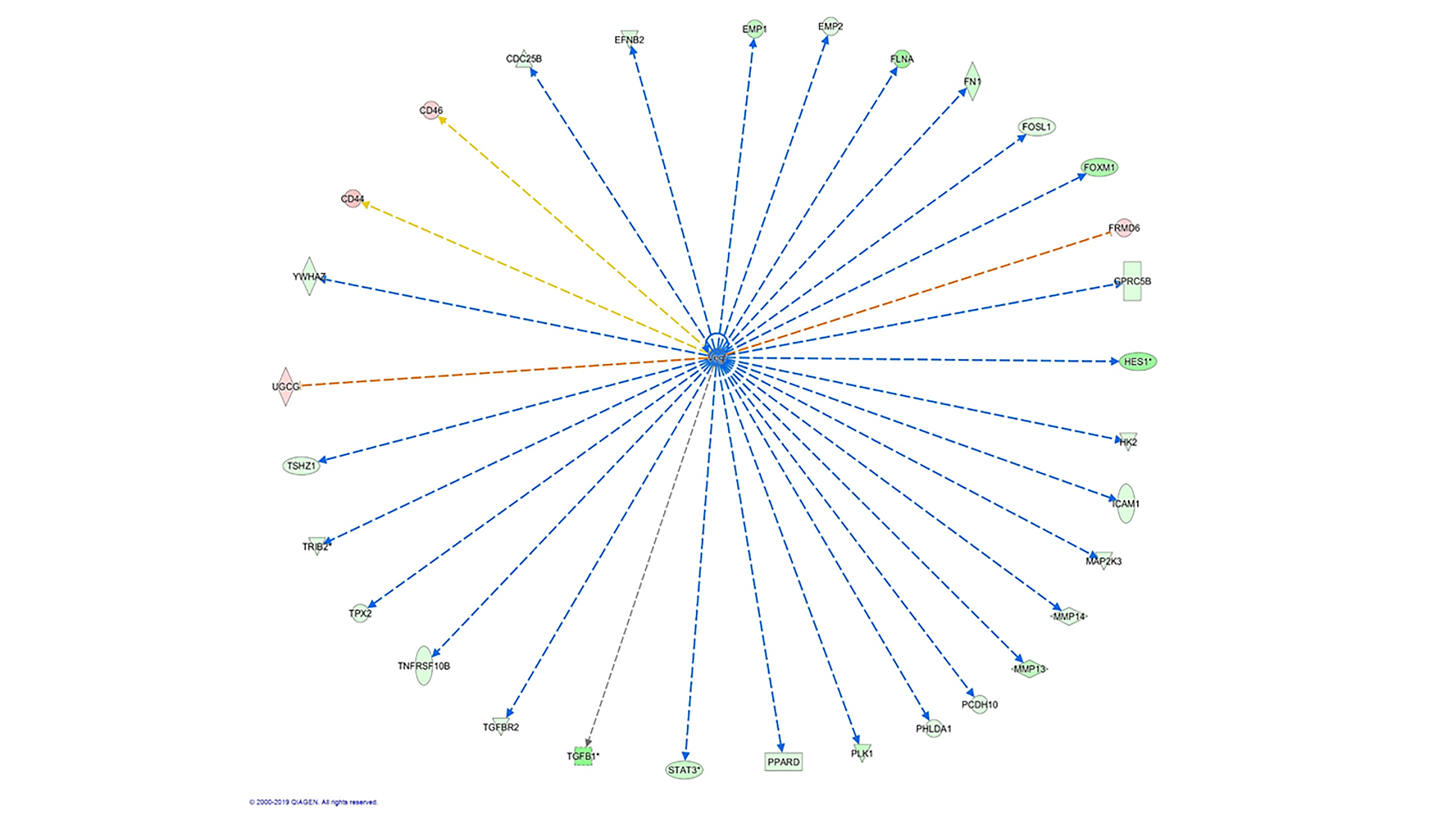

Supplement: Supplementary file 7 — Supplementary Figure 4 [file 41418_2020_505_MOESM7_ESM.tif]

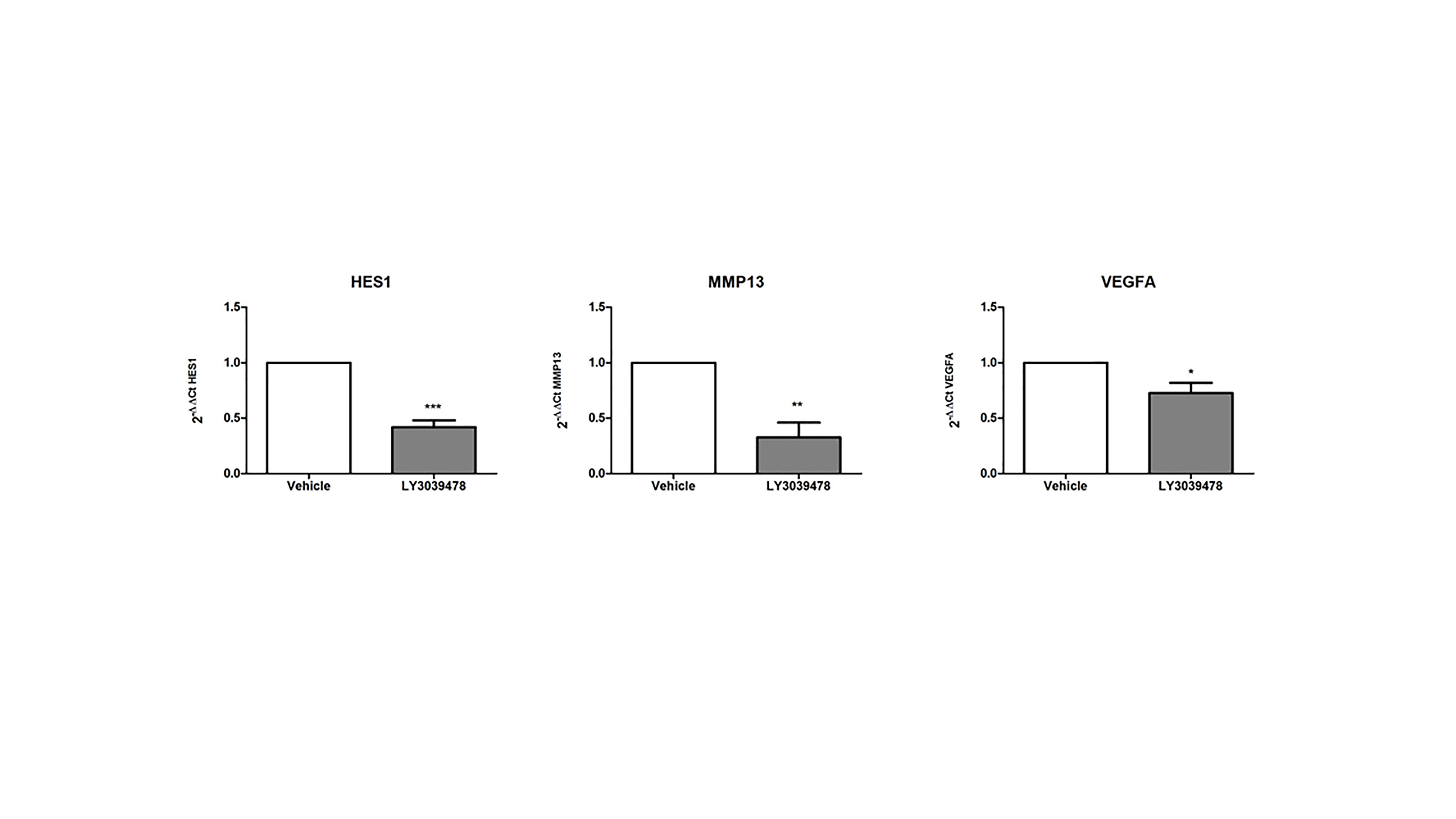

Supplement: Supplementary file 8 — Supplementary Figure 5 [file 41418_2020_505_MOESM8_ESM.tif]

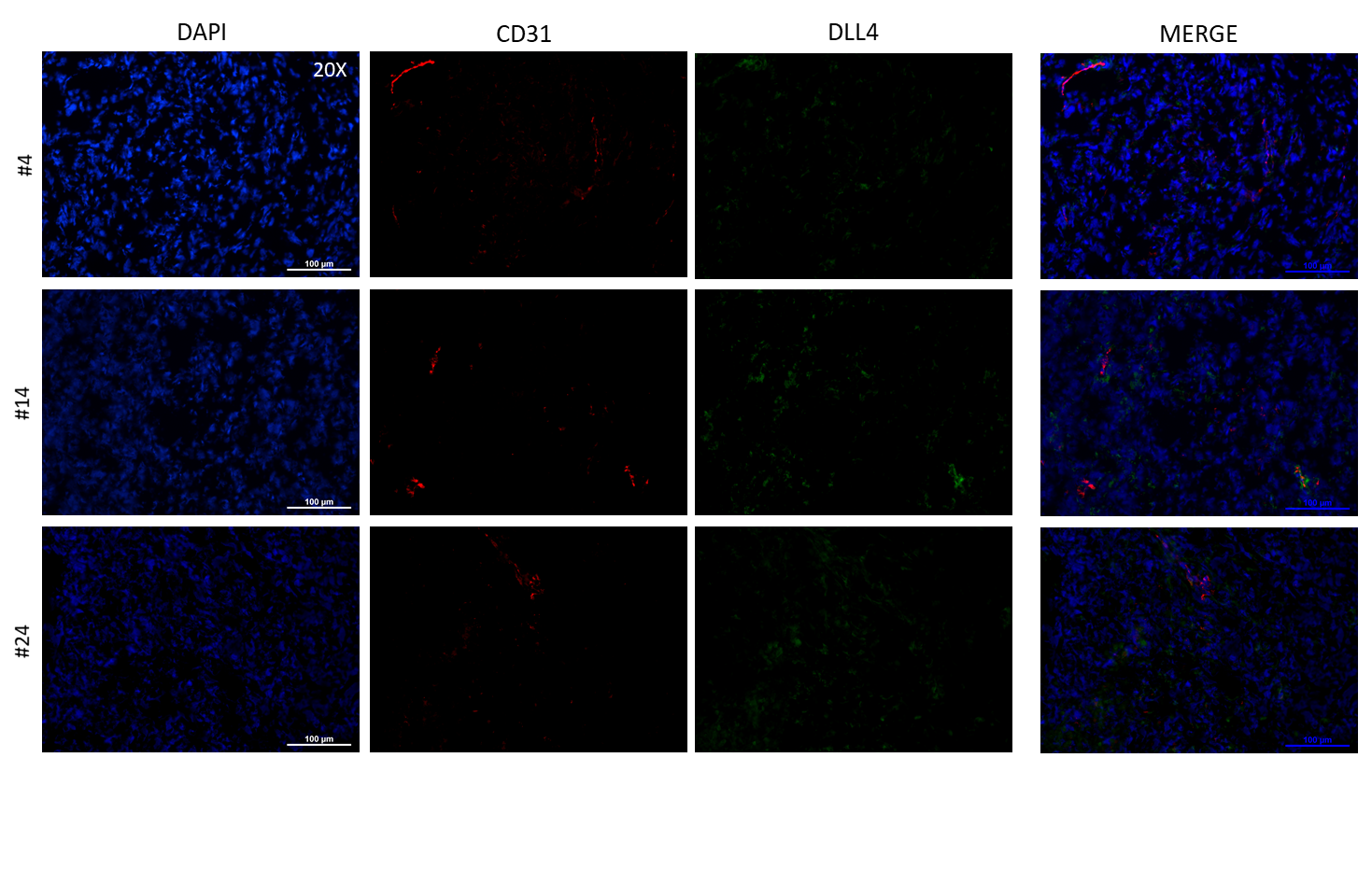

Supplement: Supplementary file 9 — Supplementary Figure 6 [file 41418_2020_505_MOESM9_ESM.tif]

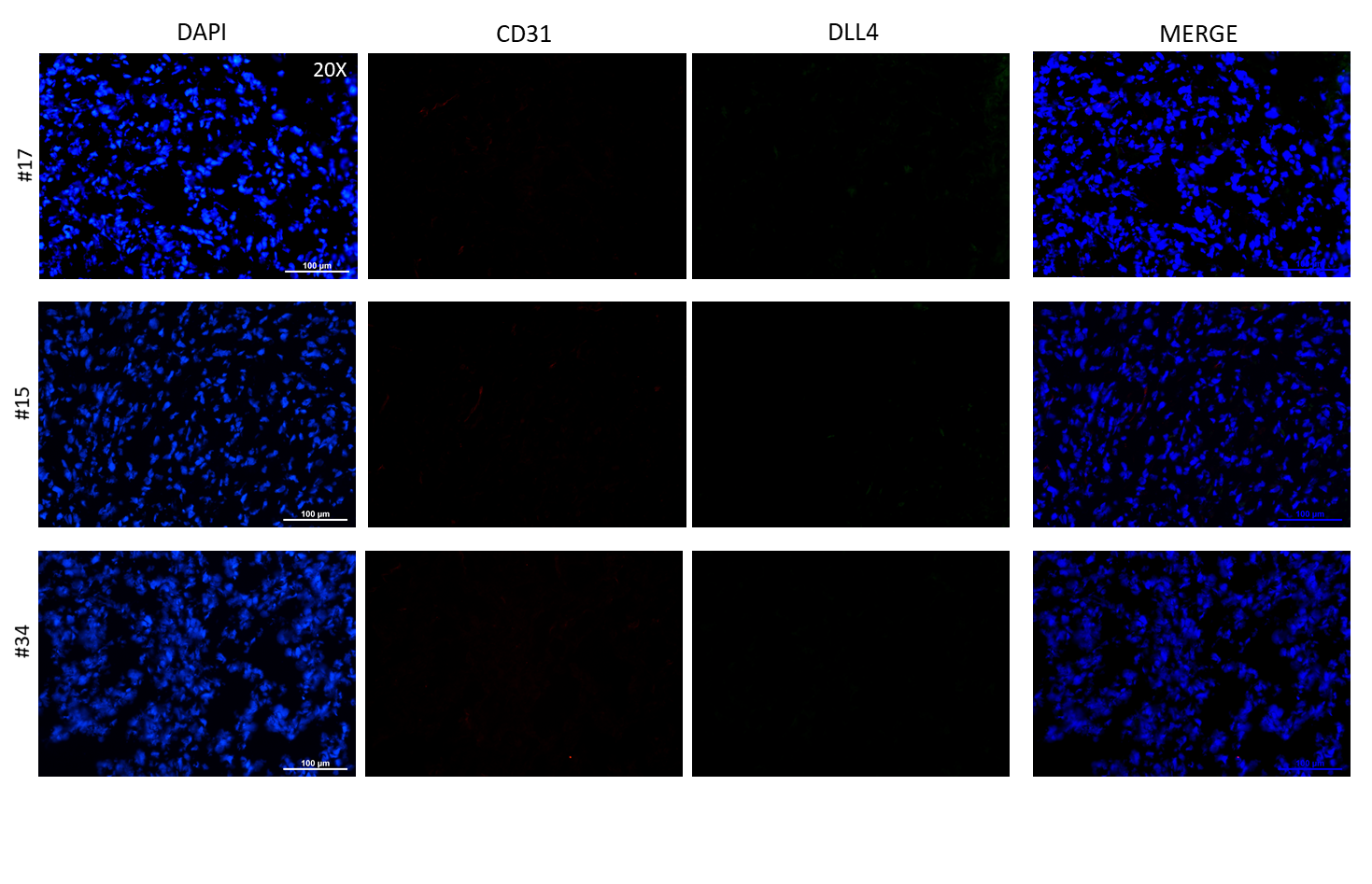

Supplement: Supplementary file 10 — Supplementary Figure 7 [file 41418_2020_505_MOESM10_ESM.tif]

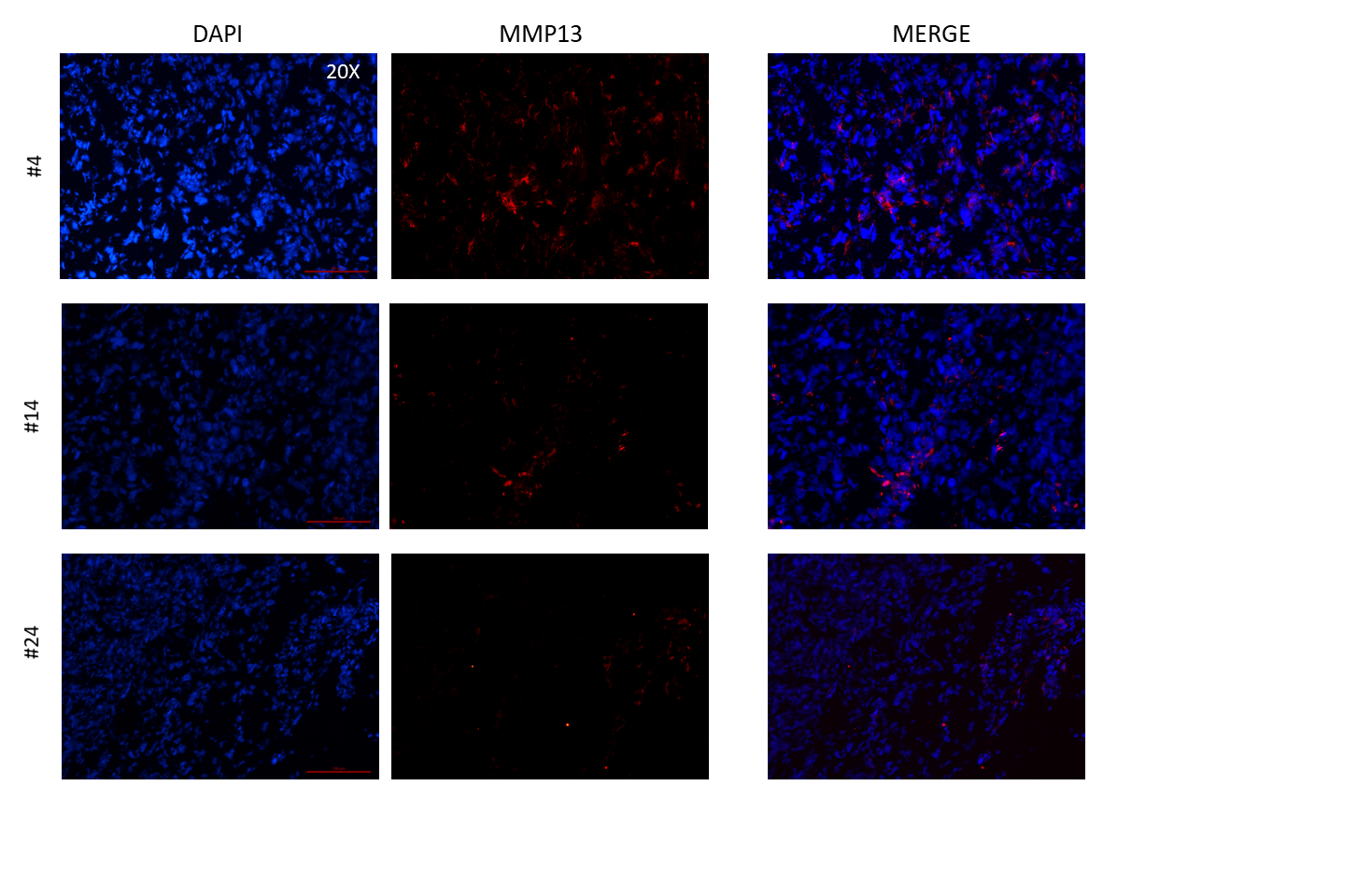

Supplement: Supplementary file 11 — Supplementary Figure 8 [file 41418_2020_505_MOESM11_ESM.tif]

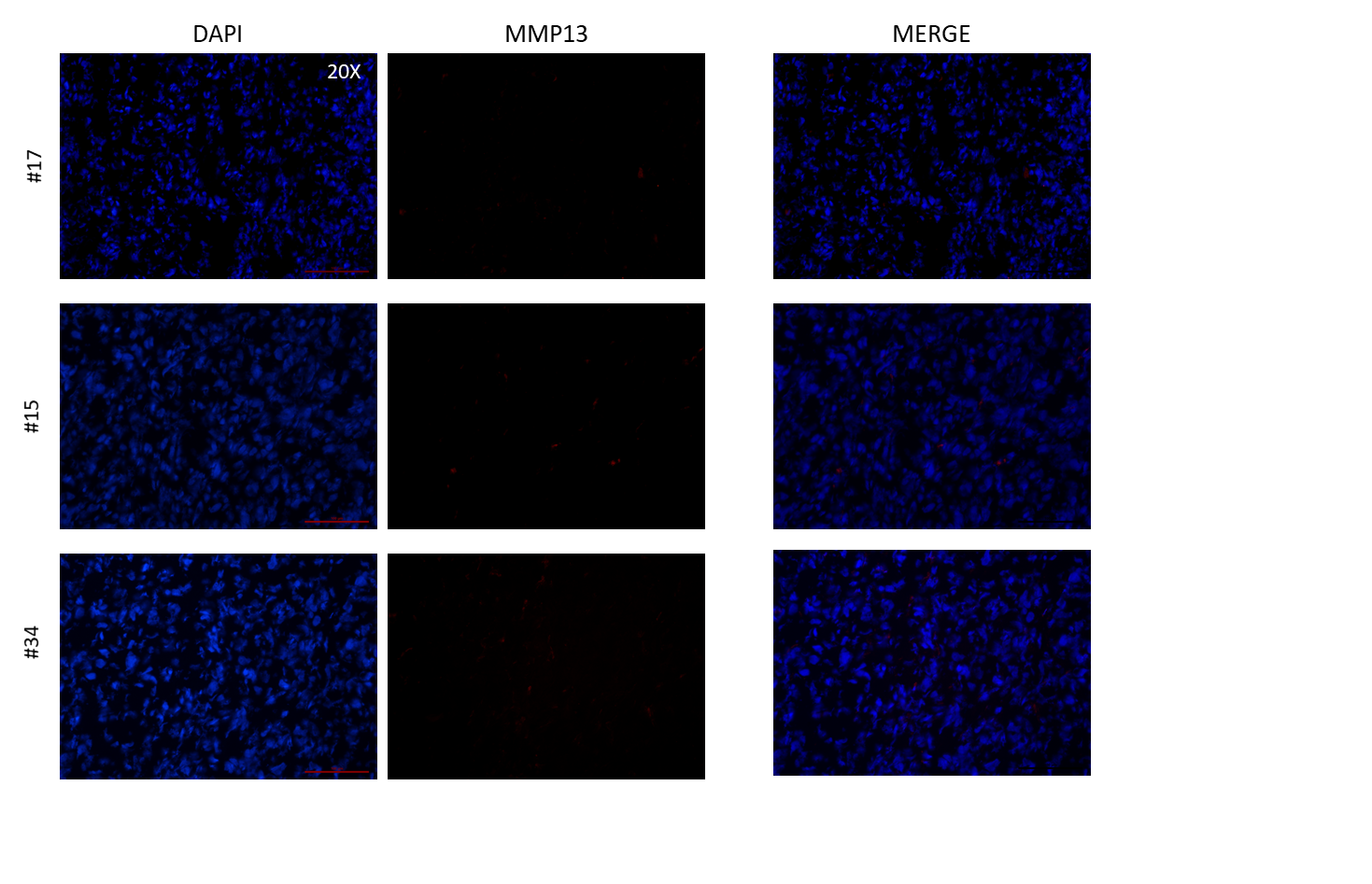

Supplement: Supplementary file 12 — Supplementary Figure 9 [file 41418_2020_505_MOESM12_ESM.tif]

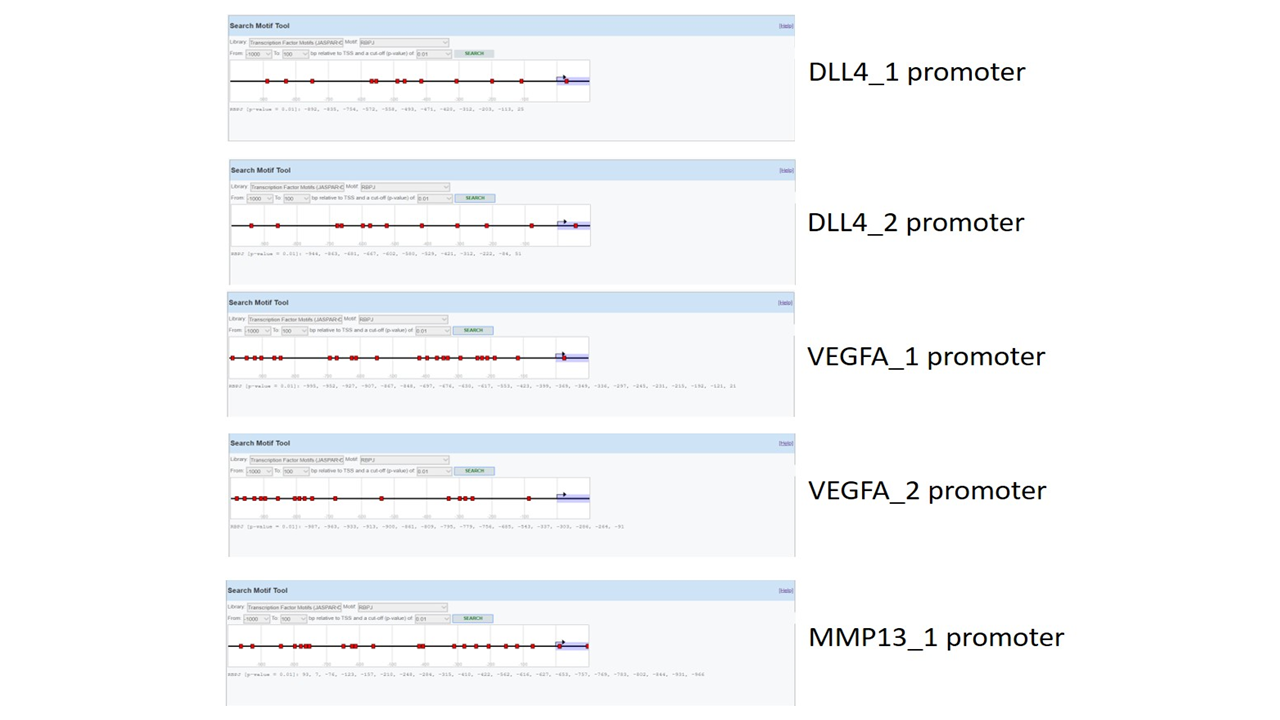

Supplement: Supplementary file 13 — Supplementary Figure 10 [file 41418_2020_505_MOESM13_ESM.tif]

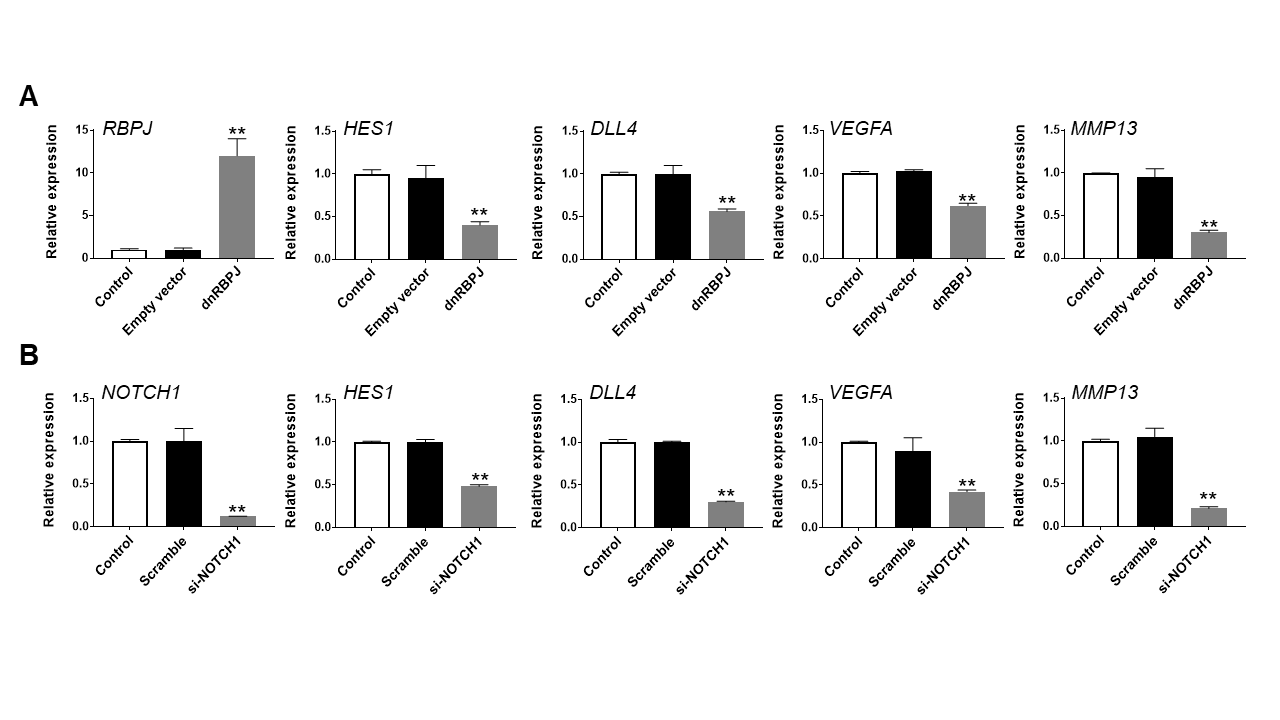

Supplement: Supplementary file 14 — Supplementary Figure 11 [file 41418_2020_505_MOESM14_ESM.tif]

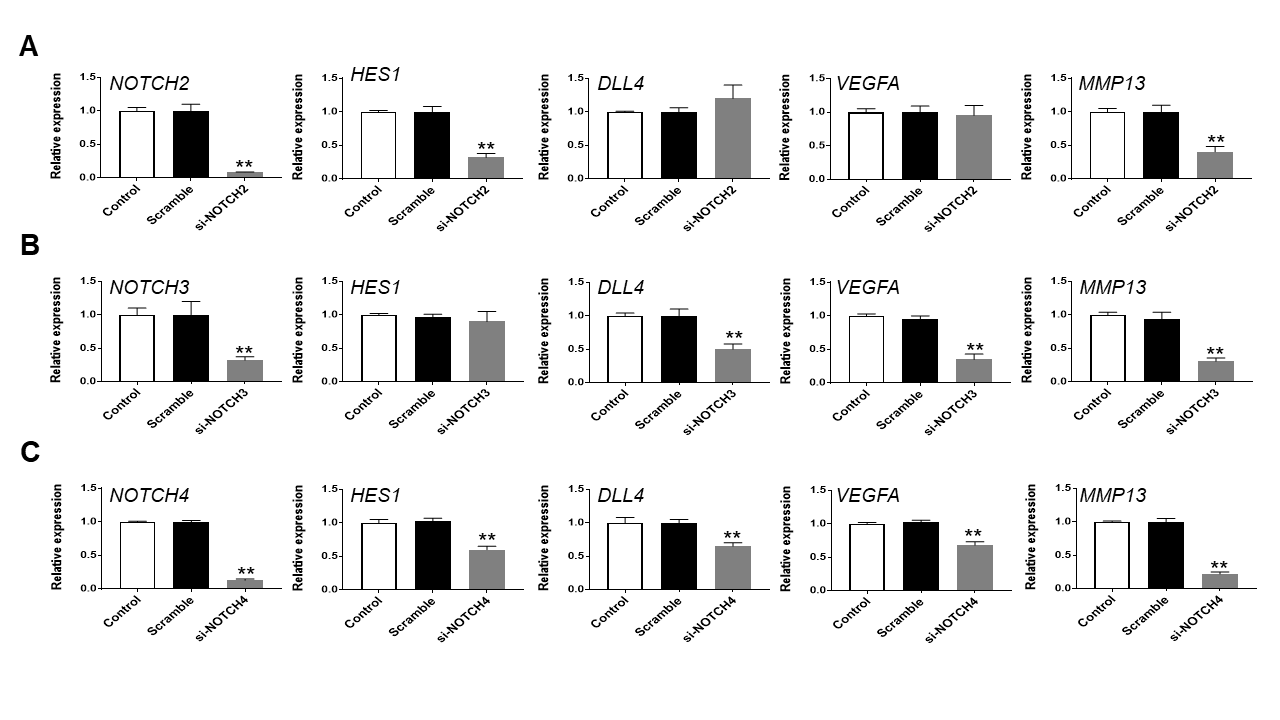

Supplement: Supplementary file 15 — Supplementary Figure 12 [file 41418_2020_505_MOESM15_ESM.tif]

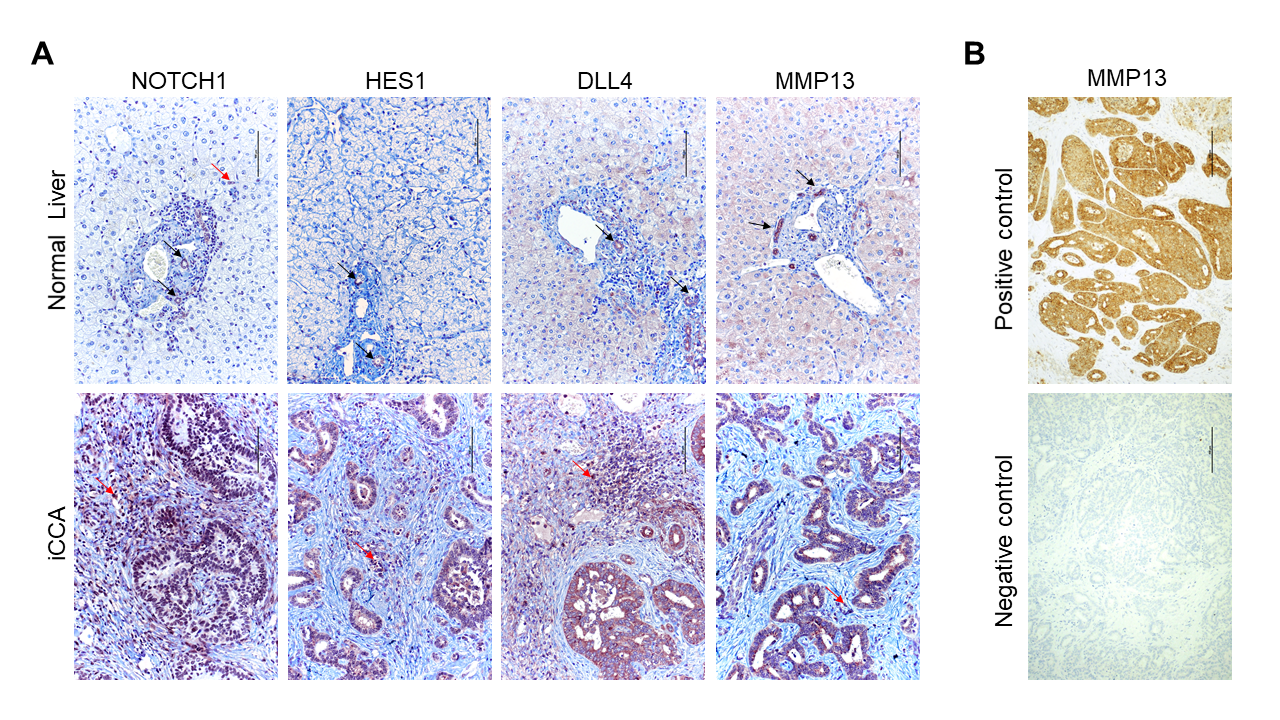

Supplement: Supplementary file 16 — Supplementary Figure 13 [file 41418_2020_505_MOESM16_ESM.tif]
