# Supplementary material for: Crenigacestat, a selective NOTCH1 inhibitor, reduces intrahepatic cholangiocarcinoma progression by blocking VEGFA/DLL4/MMP13 axis
Source: Cell Death Differ. 2020 Feb 10;27(8):2330–43. doi: 10.1038/s41418-020-0505-4 (PMC7370218; doi:10.1038/s41418-020-0505-4)
Supplement: Supplementary file 17 — Supporting information tables [file 41418_2020_505_MOESM17_ESM.docx]

Supporting informations 1. List of antibodies used for Western blot analysis

| **Antibody** | **Catalogue Number** | **Company** | **Species** | **Dilution** |
| --- | --- | --- | --- | --- |
| Anti-Notch cleaved1 (Val1744) (D3B8) | #4147 | Cell Signaling Technology, Danvers, MA, USA | Rabbit monoclonal | 1:1000 |
| Anti-Hes1 (D6P2U) | #11988 | Cell Signaling Technology, Danvers, MA, USA | Rabbit monoclonal | 1:1000 |
| Anti-VEGFA[VG-1] | Ab1316 | Abcam, Cambridge, MA | Mouse monoclonal | 1:1000 |
| Anti-DLL4 | Ab7280 | Abcam, Cambridge, MA | Rabbit polyclonal | 1:1000 |
| Anti-CD31 | Ab28364 | Abcam, Cambridge, MA | Rabbit polyclonal | 1:1000 |
| Anti-GAPDH | WH0002597M1 clone 3C2M | Merk Millipore, Burlington, MA, US) | Mouse monoclonal | 1:1000 |
| Anti Rabbit IgG HRP Linked Ab | #7074 | Cell Signaling Technology, Danvers, MA, USA |  | 1:5000 |
| Anti Mouse IgG HRP Linked Ab | #7076 | Cell Signaling Technology, Danvers, MA, USA |  | 1:5000 |

Supporting informations 2. List of antibodies used for Immunohistochemistry and Immunofluorescence

| **Antibody** | **Catalogue Number** | **Company** | **Species** | **Dilution** |
| --- | --- | --- | --- | --- |
| Anti-CK19 | ab15463 | Abcam, Cambridge, MA | Rabbit polyclonal | 1:50 |
| Anti-CK18 [C-04] | (ab668) | Abcam, Cambridge, MA | Mouse monoclonal | 1:50 |
| Anti-CK7 [EPR17078] | ab181598 | Abcam, Cambridge, MA | Rabbit monoclonal | 1:50 |
| Anti-Actin (C4) | sc-47778 | Santa Cruz Biothecnology, Inc., Heidelberg Germany | Mouse monoclonal | 1:50 |
| Anti-Vimentin (D21H3) | #5741 | Cell Signaling Technology, Danvers, MA, USA | Rabbit monoclonal | 1:50 |
| Anti-Ki67 | MA5-14520 | ThermoFisher Scientific, Waltham, MA | Rabbit monoclonal | 1:250 |
| Anti-CD31 | Ab28364 | Abcam, Cambridge, MA | Rabbit polyclonal | 1:50 |
| Anti-DLL4 (G-12) | Sc-365429 | Santa Cruz Biothecnology, Inc., Heidelberg Germany | Mouse monoclonal | 1:50 |
| Anti-MMP13 | Ab39012 | Abcam, Cambridge, MA | Rabbit polyclonal | 1:50 |
| anti-CD34 | Ab110643 | Abcam, Cambridge, MA | Rabbit molyclonal | 1:2000 |

Supporting informations 3. List of the cell lines used for the experiments

| **Cell lines** | **Cell line type** | **Culture Condition** |
| --- | --- | --- |
| HUCCT-1 | Human iCCA | RPM1+10%FBS+1%Anti anti |
| KMCH1 | Human iCCA | DMEM+10%FBS+1%Anti anti |
| RBE | Human iCCA | RPM1+10%FBS+1%Anti anti |
| KKU-M123 | Human iCCA | DMEM+10%FBS+1%Anti anti |
| KKU-M156 | Human iCCA | DMEM+10%FBS+1%Anti anti DMEM+10%FBS+1%Anti anti |
| HUVEC | Human iCCA | EndoGRO-LS Complete Culture Media Kit |

Supporting informations 4. Clinical characteristics of the patients (NA = not available, PSC = primary sclerosing cholangitis)

| **#** | **Age** | **Sex** | **Cirrhosis** | **Etiology** | **Tumor size** | **Tumor differentiation** | **Tumor number** | **Lymph node metastasis** |
| --- | --- | --- | --- | --- | --- | --- | --- | --- |
| **1** | 62 | M | Yes | HCV | > 3cm | Well-differentiated | Single | NO |
| **2** | 48 | M | NO | NA | > 3cm | Well-differentiated | Single | NO |
| **3** | 58 | M | NO | HBV | > 3cm | Moderately-differentiated | Multiple | NO |
| **4** | 42 | F | Yes | HBV | < 3 cm | Well-differentiated | Multiple | Yes |
| **5** | 70 | M | Yes | HBV | < 3 cm | Moderately-differentiated | Multiple | Yes |
| **6** | 56 | M | NO | NA | > 3cm | Well-differentiated | Single | NA |
| **7** | 40 | F | NO | Hepatolithiasis | > 3cm | Well-differentiated | Single | NO |
| **8** | 88 | M | NO | Hepatolithiasis | > 3cm | Moderately-differentiated | Multiple | NO |
| **9** | 78 | F | NO | HBV | > 3cm | Moderately-differentiated | Single | NO |
| **10** | 50 | F | Yes | NA | > 3cm | Poorly-differentiated | Single | Yes |
| **11** | 72 | M | NO | PSC | < 3 cm | Well-differentiated | Single | NO |
| **12** | 68 | M | Yes | HCV | > 3cm | Moderately-differentiated | Single | NA |
| **13** | 62 | M | NO | HCV | > 3cm | Well-differentiated | Single | NO |
| **14** | 60 | M | NO | Hepatolithiasis | < 3 cm | Well-differentiated | Single | NA |
| **15** | 84 | F | Yes | Ethanol | > 3cm | Moderately-differentiated | Multiple | NO |
| **16** | 76 | M | NO | Ethanol | > 3cm | Well-differentiated | Multiple | Yes |
| **17** | 79 | M | NO | HBV | < 3 cm | Moderately-differentiated | Single | NA |
| **18** | 48 | M | NO | NA | < 3 cm | Moderately-differentiated | Single | NO |
| **19** | 55 | M | NO | Hepatolithiasis | > 3cm | Well-differentiated | Multiple | N |
| **20** | 70 | F | NO | Hepatolithiasis | > 3cm | Moderately-differentiated | Multiple | Yes |
| **21** | 44 | M | Yes | HBV | > 3cm | Poorly-differentiated | Single | NA |
| **22** | 56 | F | Yes | HCV | < 3 cm | Well-differentiated | Single | NA |
| **23** | 72 | M | NO | NA | > 3cm | Well-differentiated | Single | NA |
| **24** | 66 | M | NO | Hepatolithiasis | > 3cm | Moderately-differentiated | Single | NA |
| **25** | 61 | F | NO | Hepatolithiasis | > 3cm | Poorly-differentiated | Single | NO |
| **26** | 58 | M | Yes | HBV | < 3 cm | Well-differentiated | Single | Yes |
| **27** | 54 | M | Yes | HCV | > 3cm | Well-differentiated | Multiple | NA |
| **28** | 72 | M | Yes | HBV | < 3 cm | Poorly-differentiated | Single | NA |
| **29** | 48 | F | NO | Hepatolithiasis | < 3 cm | Well-differentiated | Single | NA |
| **30** | 62 | F | Yes | HBV | > 3cm | Moderately-differentiated | Single | NO |
| **31** | 76 | M | NO | HCV | < 3 cm | Well-differentiated | Single | NA |
| **32** | 81 | M | NO | PSC | > 3cm | Moderately-differentiated | Single | NA |
| **33** | 63 | M | NO | NA | > 3cm | Moderately-differentiated | Single | NO |
| **34** | 68 | F | Yes | Hepatolithiasis | > 3cm | Well-differentiated | Single | NA |
| **35** | 75 | F | Yes | HBV | > 3cm | Well-differentiated | Single | NO |
| **36** | 62 | F | NO | Hepatolithiasis | < 3 cm | Moderately-differentiated | Single | NA |
| **37** | 52 | M | NO | Hepatolithiasis | > 3cm | Well-differentiated | Multiple | NO |
| **38** | 71 | M | NO | NA | > 3cm | Well-differentiated | Single | NA |
| **39** | 70 | F | NO | HBV | > 3cm | Well-differentiated | Single | NO |
| **40** | 56 | M | NO | NA | > 3cm | Poorly-differentiated | Single | NA |
| **41** | 80 | M | NO | NA | > 3cm | Moderately-differentiated | Multiple | NO |
| **42** | 62 | M | Yes | HBV | > 3cm | Well-differentiated | Single | NO |
| **MEAN** | **63,69** |  |  |  |  |  |  |  |
| **POP.**  **ST.**  **DEV.** | **11,74** |  |  |  |  |  |  |  |
| **SEM** | **3,91** |  |  |  |  |  |  |  |
